# Acute effects of 3 and 6 mg·Kg^−1^ caffeine on strength–power performance and isokinetic peak torque in competitive male boxers: a randomized, double-blind, placebo-controlled crossover trial

**DOI:** 10.1080/15502783.2026.2743368

**Published:** 2026-10-07

**Authors:** Maiwulanjiang Tuerhongjiang, Zhijie Yan, Hao Wang, Zichen Zeng, Ruidong Liu

**Affiliations:** a Sports Coaching College, Beijing Sport University, Beijing, China; b Key Laboratory of Sport Training of General Administration of Sport of China, Beijing Sport University, Beijing, China

**Keywords:** Caffeine ingestion, male boxers, strength–power performance, countermovement jump, ballistic push-up, isokinetic peak torque

## Abstract

**Background:**

Evidence on the dose, timing, and task specificity of caffeine ingestion in combat-sport athletes remains limited. This study examined the acute effects of 3 and 6 mg·kg^−1^ caffeine on strength–power performance and isokinetic peak torque in male boxers.

**Methods:**

Fifteen male boxers who met the Chinese national second-class athlete standard or higher completed a randomized, double-blind, placebo-controlled crossover trial. Each participant completed placebo (PLA), 3 mg·kg^−1^ caffeine (CAF3), and 6 mg·kg^−1^ caffeine (CAF6) conditions in randomized order, separated by a 1-week washout. Countermovement jump (CMJ), ballistic push-up (BPU), and dominant-side shoulder and knee isokinetic peak torque (PT) were assessed at baseline and during testing blocks beginning at 30 and 60 min post-ingestion. Data were analyzed using two-way repeated-measures ANOVA, with Holm adjustment applied across the 10 planned condition × time interaction tests. Reliability analyzes were supportive, and threshold-based individual classifications were exploratory.

**Results:**

Significant condition × time interactions were observed for CMJ, BPU, and isokinetic PT outcomes. During the scheduled 60-min assessment, CAF3 and CAF6 produced higher CMJ jump height, peak power, and relative peak power than PLA, with no significant difference between caffeine doses. For BPU, CAF6 produced higher concentric impulse, concentric mean power, and concentric peak power than PLA during the scheduled 60-min assessment, whereas CAF3 did not differ from PLA. Shoulder flexion and extension PT increased under caffeine conditions at selected time points, without a consistent dose advantage. Knee extension PT showed the clearest dose-specific response, with CAF6 exceeding both PLA and CAF3 during the scheduled 60-min assessment.

**Conclusions:**

Acute ingestion of 3 and 6 mg·kg^−1^ caffeine was associated with improvements in selected laboratory-based strength–power and isokinetic PT outcomes in male boxers, particularly during the scheduled 60-min assessment. The pattern of responses varied across doses and testing tasks, and 6 mg·kg^−1^ was not consistently superior to 3 mg·kg^−1^. These findings should be interpreted as evidence for selected neuromuscular outcomes rather than as direct evidence of improved punching or competitive boxing performance.

## 1. Introduction

Boxing is characterized by repeated high-intensity offensive and defensive actions, rapid footwork, and the need to generate force within short time intervals across multiple rounds [1]. Although the punch is expressed through the upper limb, effective striking is a whole-body kinetic-chain action in which force is generated from the lower limbs, transferred through the trunk, and ultimately expressed through the shoulder, elbow, and wrist [2,3]. Consequently, lower-limb explosive capacity, upper-body ballistic force production, and joint-specific strength may represent important physical qualities supporting boxing performance. Previous work in boxers has shown associations between strength–power qualities and punching impact, and isokinetic characteristics of the knee and shoulder have also been examined in relation to boxing-specific strength performance [3–5]. Within this context, the countermovement jump (CMJ) provides an index of lower-limb explosive performance, the ballistic push-up (BPU) reflects upper-body closed-chain ballistic pushing capacity, and isokinetic peak torque (PT) provides a standardized measure of joint-specific maximal torque production. However, these laboratory-based tests do not reproduce the open-chain, rotational, technically constrained nature of punching and should therefore be interpreted as boxing-relevant neuromuscular measures rather than direct measures of punching or competitive boxing performance.

Caffeine is among the most widely studied acute ergogenic aids in sport nutrition. Current consensus and position statements identify caffeine as a supplement with evidence-based performance benefits when it is used appropriately [6,7]. At doses commonly used in sport, caffeine primarily acts as an adenosine-receptor antagonist, which may reduce adenosine-mediated inhibition and influence arousal, perceived effort, voluntary activation, and neuromuscular performance [7–10]. For strength performance, these effects may facilitate voluntary neural drive during maximal contractions, while peripheral effects on excitation–contraction processes have also been proposed but are less clearly established at typical doses. Doses of 3–6 mg·kg^−1^, typically ingested about 60 min before exercise, are widely used in sport practice [7]. The timing of caffeine ingestion may also influence the ergogenic response. Following ingestion of encapsulated caffeine, plasma caffeine concentrations increase progressively, while the time to peak concentration can vary considerably among individuals and may extend beyond the first hour [11]. Accordingly, assessment at 30 min may capture an earlier phase of caffeine absorption and performance response, whereas approximately 60 min represents a more commonly used pre-exercise interval [7,12]. Interindividual responses may also be influenced by genetic variation related to caffeine metabolism, including CYP1A2 polymorphisms [13]. Previous studies indicate that caffeine can improve maximal strength, isokinetic strength, vertical jumping, and power output, although null findings have also been reported for some jump and power outcomes [13–16]. These mixed results suggest that caffeine responses may depend on dose, timing, training background, and the specific testing task.

Whether the ergogenic effects of caffeine are expressed similarly across upper- and lower-body tasks remains unclear. Previous evidence suggests that acute caffeine ingestion can improve both upper- and lower-body strength and power, although the magnitude of these responses has not been consistent across studies. Recent meta-analytic evidence indicates beneficial effects on both upper- and lower-body muscle strength, with some analyzes suggesting greater effects for lower-body strength, while direct within-participant studies have also reported differences in the magnitude of caffeine-related responses between upper- and lower-body exercises [9,14,17]. These inconsistencies may reflect differences in exercise mode, muscle mass, movement complexity, training status, and measurement characteristics. The dose-response relationship is similarly uncertain. Although 3–6 mg·kg^−1^ is a commonly used ergogenic range, direct dose-comparison studies indicate that increasing caffeine intake does not necessarily produce proportionally greater improvements in strength or power, and that any additional benefit may depend on the exercise task and loading condition [18]. Therefore, direct within-participant comparisons of 3 and 6 mg·kg^−1^ caffeine across multiple upper- and lower-body neuromuscular tasks may help clarify whether caffeine responses are dose- and task-dependent.

In combat sports, caffeine responses may be particularly task-dependent because performance depends on both general neuromuscular capacity and sport-specific technical execution. Previous studies in combat-sport athletes have examined caffeine-related changes in physical performance, perceived exertion, and technical-tactical or discipline-specific performance, with responses varying across sports, doses, and testing protocols [19–22]. Evidence specifically in boxers remains limited. Nevertheless, strength–power qualities and knee and shoulder strength have been associated with punching-related performance in boxers [3–5], supporting the relevance of the neuromuscular outcomes examined here. Evidence remains especially limited in boxers. It is still unclear whether commonly used caffeine doses produce similar effects across lower-limb explosive tasks, upper-limb ballistic pushing tasks, and isolated joint-strength measures. It is also unclear whether responses are already evident during the scheduled 30-min assessment or are more pronounced during the scheduled 60-min assessment, the latter reflecting the timing most often used in practice.

Therefore, this randomized, double-blind, placebo-controlled crossover trial directly compared the acute effects of PLA, 3 mg·kg^−1^ caffeine, and 6 mg·kg^−1^ caffeine across lower-limb explosive performance (CMJ), upper-limb ballistic performance (BPU), and upper- and lower-body joint-specific isokinetic PT in competitive male boxers. Performance was assessed at baseline and during testing blocks beginning at 30 and 60 min post-ingestion to determine whether caffeine responses varied according to dose, post-ingestion timing, and testing task. We hypothesized that caffeine would improve selected strength–power and isokinetic outcomes, but that the magnitude of the response would vary across doses, time points, and testing tasks.

## 2. Methods

### 2.1. Study design

This study used a randomized, double-blind, placebo-controlled crossover design. The three supplementation conditions were placebo (PLA), 3 mg·kg^−1^ caffeine (CAF3), and 6 mg·kg^−1^ caffeine (CAF6). Each participant completed all three conditions, separated by a 1-week washout. Each formal trial followed the same sequence: standardized warm-up, baseline testing, supplement ingestion, and repeated testing during blocks beginning at 30 and 60 min post-ingestion. Outcomes included CMJ, BPU, and dominant-side shoulder and knee isokinetic PT. Additional details on the testing protocol and counterbalanced supplementation sequences are provided in Supplementary Tables S1 and S2.

### 2.2. Participants

An a priori sample-size estimation was conducted using GPower 3.1 [23]. The effect-size assumption (f = 0.30) was informed by Harty et al. [12], who reported effects of a similar magnitude for caffeine-related changes in lower-body neuromuscular performance. With *α* = 0.05 and statistical power of 1 − *β* = 0.80, the original calculation indicated that at least 12 participants were required. Allowing for an anticipated dropout rate of approximately 20%, 15 participants were recruited. The calculation was used as a general repeated-measures planning estimate and was not specifically powered for the condition × time interaction. All participants completed the PLA, CAF3, and CAF6 conditions and were included in the final analysis. Participants were recruited in May 2025 at Beijing Sport University, Beijing, China. Participant characteristics are shown in Table 1.

The inclusion criteria were age 18–30 years, Chinese national second-class athlete standard or higher in boxing, systematic boxing training experience >3 years, no cardiovascular risk, no musculoskeletal injury or other contraindication to exercise, no history of smoking or alcohol abuse, no known adverse reaction to caffeine, and no concurrent participation in any other research study during the study period. Before enrollment, all participants were informed about the study procedures, testing content, and precautions, and all provided written informed consent. To minimize external influences, participants were instructed to avoid coffee, tea, milk tea, energy drinks, and other caffeine-containing foods or beverages for 48 h before each formal trial and to avoid alcohol and smoking. The 48-h abstinence period was used to standardize recent caffeine exposure across experimental conditions rather than because this duration was considered pharmacokinetically necessary. Habitual caffeine consumption was not systematically quantified before enrollment. They were also instructed to avoid strenuous exercise for 24 h before testing. Before each formal trial, participants were asked to maintain similar dietary intake and sleep routines. Each participant completed all formal trials at approximately the same time of day. Depending on individual scheduling, experimental trials were conducted primarily in the morning or afternoon. The timing of the last meal was not standardized; however, participants did not consume food during the 1 h immediately preceding each trial. Participants were asked to maintain a similar pre-trial dietary pattern across conditions. All trials used the same testing order, standardized warm-up, rest intervals, and verbal instructions. The study was approved by the Sports Science Experimental Ethics Committee of Beijing Sport University (approval No. 2025551 H) and was conducted in accordance with the Declaration of Helsinki.

**Table 1.** Participant characteristics.

Variable	Mean ± SD
Age, years	21.53 ± 1.96
Height, cm	178.13 ± 1.96
Body mass, kg	74.00 ± 7.68
Training experience, years	4.13 ± 1.30
Fat mass, kg	12.57 ± 3.45
Lean body mass, kg	61.43 ± 4.37

Note: Values are mean ± SD.

### 2.3. Supplementation protocol, randomization, and blinding

The study included PLA, CAF3, and CAF6 conditions. The number, appearance, and packaging of capsules were identical across conditions. Placebo capsules were filled with maltodextrin, whereas CAF3 and CAF6 capsules contained the required caffeine dose plus maltodextrin to match capsule number and filling volume across conditions. Caffeine was obtained from Nutricost Caffeine Capsules (Nutricost, USA). Before testing, the required caffeine dose for CAF3 and CAF6 was calculated according to each participant’s body mass and weighed using a precision electronic balance (Sartorius Quintix125D-1CN, Sartorius, Germany). The placebo consisted of Organic Maltodextrin (Nutricost, USA), which contained no caffeine or other stimulant ingredients. Caffeine powder and maltodextrin were placed in identical capsules and packed in sealed coded envelopes. During each formal trial, the assigned capsules were ingested with 200 mL of water immediately after baseline testing. Weighing, encapsulation, coding, and distribution were completed by an investigator who was not involved in testing or data analysis, thereby maintaining allocation concealment and blinding.

Randomization was performed using a counterbalanced Latin-square design. The three supplementation sequences were PLA–CAF3–CAF6, CAF3–CAF6–PLA, and CAF6–PLA–CAF3. The 15 participants were randomly allocated to these sequences in a 1:1:1 ratio (*n* = 5 per sequence), ensuring that each supplementation condition appeared five times in the first, second, and third formal trials. This counterbalancing was used to reduce potential imbalance related to treatment order and study period, rather than to demonstrate the absence of period or sequence effects. The randomization sequence was generated and stored by the independent investigator described above. Participants, testers, and data analysts remained blinded to the supplementation allocation until statistical analyzes had been completed. During each formal trial, participants were verbally asked whether they experienced any symptoms potentially related to supplementation. However, symptom type, frequency, and severity were not systematically recorded using a standardized questionnaire, predefined symptom checklist, or severity-rating scale. Blinding success was not formally assessed, and participants were not asked to guess their supplementation condition. Formal trials were separated by a 1-week washout to reduce the potential for residual treatment and carryover effects. However, period, sequence, and carryover effects were not formally tested. The counterbalanced sequence structure is summarized in Supplementary Table S2.

### 2.4. Experimental procedure

Each participant completed four laboratory visits, comprising one familiarization session and three formal experimental trials (Figure 1). The familiarization session included body-composition assessment, an explanation of the experimental procedures, and practice of all testing movements. At the beginning of each formal trial, participants completed a standardized 10-min warm-up consisting of 5 min of treadmill jogging at 8 km·h^−1^, followed by 5 min of upper- and lower-body dynamic mobility exercises. The upper-body sequence comprised bilateral forward and backward shoulder circles, single-arm large arm circles, and dynamic shoulder internal–external rotations performed with the shoulder abducted to approximately 90° and the elbow flexed to approximately 90°. The lower-body sequence comprised the world’s greatest stretch, dynamic lateral lunges, and 90/90 hip rotations. Each exercise was performed for two sets of 10 repetitions; unilateral and side-specific exercises were completed on both sides. The exercise order, range of motion, and volume were kept consistent across all experimental conditions. Participants then completed baseline testing in a fixed order: CMJ, BPU, shoulder isokinetic peak torque, and knee isokinetic peak torque. Immediately after baseline testing, participants ingested the assigned capsules with 200 mL of water. No additional caffeine-containing foods or beverages were permitted during the formal trial. The 30- and 60-min assessments were selected to compare an earlier post-ingestion response with the more conventional ~60-min pre-exercise timing used for encapsulated caffeine [7,12]. The same fixed testing sequence was repeated in blocks beginning at 30 and 60 min post-ingestion. Each block lasted approximately 15 min; therefore, the nominal 30- and 60-min labels refer to the start of the testing blocks rather than to the exact measurement time of every outcome. Consequently, outcomes were assessed approximately within 30–45 min and 60–75 min post-ingestion, respectively, with the force-platform tests occurring earlier in each block and the shoulder and knee isokinetic assessments occurring later. Exact outcome-specific timestamps were not prospectively recorded. The test order and duration were kept consistent across conditions. Outside the testing periods, participants remained seated and refrained from unnecessary physical activity until the next assessment.

**Figure 1.**
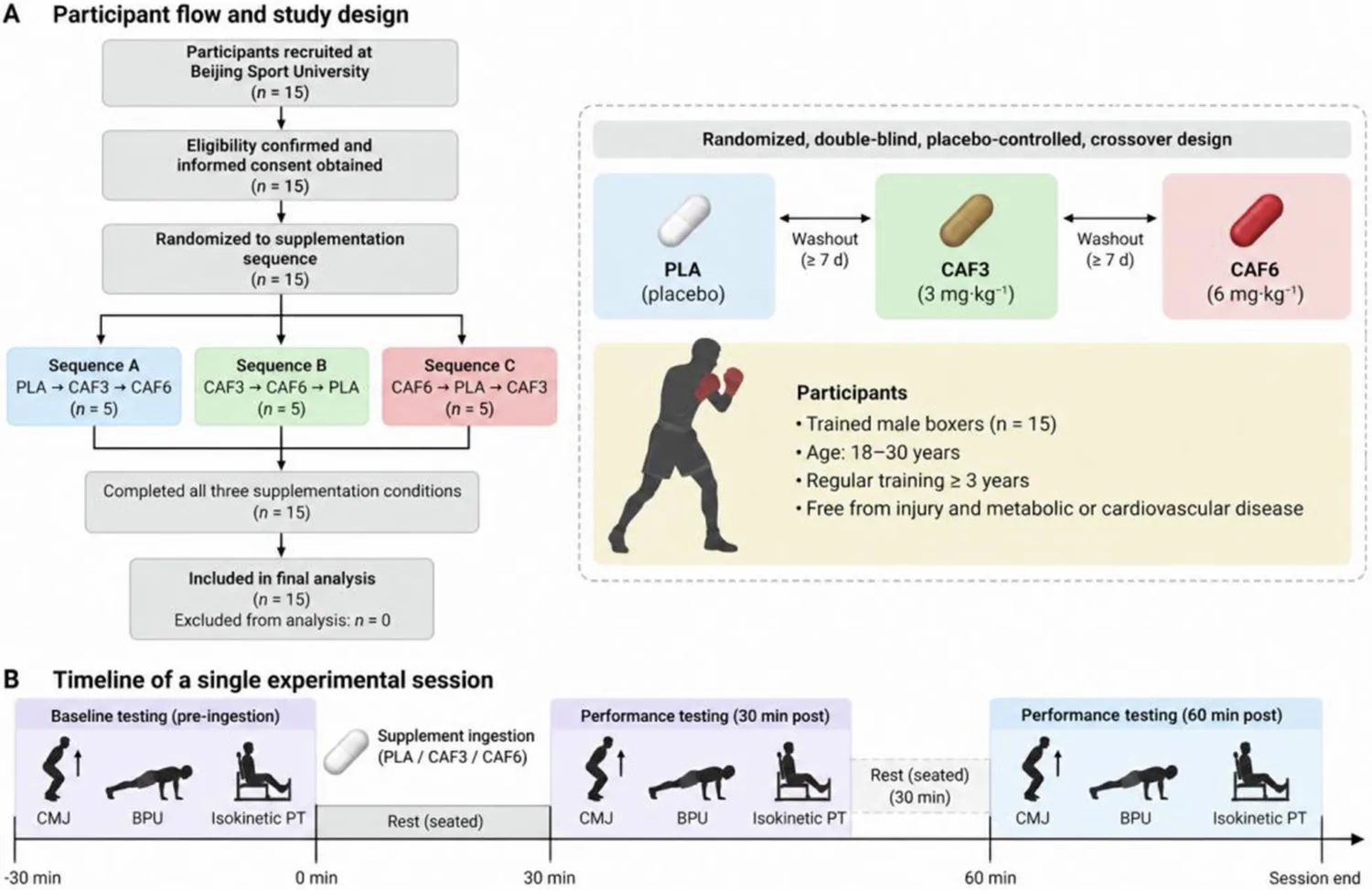
Participant flow, study design, and experimental procedure. (A) Participant recruitment, eligibility confirmation, randomization to the three counterbalanced supplementation sequences, study completion, and final analysis. Fifteen participants were randomized equally to Sequence A (PLA → CAF3 → CAF6), Sequence B (CAF3 → CAF6 → PLA), or Sequence C (CAF6 → PLA → CAF3) (*n* = 5 per sequence). All participants completed the three supplementation conditions and were included in the final analysis. Experimental conditions were separated by a ≥7-day washout period. (B) Timeline of a single experimental session, including standardized preparation, baseline testing, supplement ingestion, seated rest, and post-ingestion performance-testing blocks beginning at 30 and 60 min. CMJ, countermovement jump; BPU, ballistic push-up; PT, peak torque; PLA, placebo; CAF3, 3 mg·kg^−1^ caffeine; CAF6, 6 mg·kg^−1^ caffeine.

### 2.5. Outcome measurements

CMJ was used to evaluate lower-limb vertical explosive performance, BPU was used to assess upper-limb closed-chain ballistic pushing performance, and isokinetic testing was used to evaluate shoulder and knee peak torque (PT). CMJ and BPU were performed using a portable force platform (KWFP4053, Kunwei, China) with a sampling frequency of 1000 Hz. Force-platform data were recorded and processed using the manufacturer-provided Kunwei analysis software. The force platform was zeroed and calibrated before each testing sequence in accordance with the manufacturer’s instructions. Isokinetic testing was performed using the ISOMED 2000 multi-joint isokinetic dynamometer system (D&R Ferstl GmbH, Hemau, Germany). All tests were conducted at baseline and during testing blocks beginning at 30 and 60 min post-ingestion, with identical test order, rest intervals, and verbal instructions across conditions. A concise summary of the testing protocols and scoring rules is provided in Supplementary Table S1.

The selected force-platform variables were intended to characterize global performance and mechanical output rather than provide a comprehensive force–time profile. For the CMJ, jump height, peak power, and relative peak power were used to represent vertical explosive performance and absolute and body-mass-normalized power output. For the BPU, concentric impulse, concentric mean power, and concentric peak power were used to characterize upper-body ballistic force–time and power characteristics.

For the CMJ test, participants stood in the center of the force platform with their hands placed on the hips. From an upright position, they rapidly descended to approximately 90° of knee flexion and immediately performed a maximal vertical jump while attempting to keep the trunk vertical during flight. Each participant completed five CMJs, with the first two trials used for movement preparation and the final three trials used for formal testing. Formal trials were separated by 1 min, during which participants recovered in a standing position. Jump height was derived from take-off velocity using h = v^2^/(2g), where v is take-off velocity and g = 9.81 m·s^−2^, rather than from flight time. The mean values of jump height, peak power, and relative peak power from the three formal trials were used for statistical analysis.

The BPU test was conducted according to previously published procedures [24–26] and was used to assess upper-limb closed-chain ballistic pushing performance. Before formal testing, participants watched a standardized demonstration and completed 2–3 practice trials. Participants then assumed a standard push-up position with both hands placed on the force platform, with hand spacing matched to their usual bench-press grip width. They lowered the body until the upper arms were approximately parallel to the floor and then pushed vertically as rapidly and forcefully as possible until the hands left the force platform. Participants flexed the elbows upon landing to reduce impact. Three formal trials were completed, with 1 min of recovery between trials. Vertical force–time data were processed using the manufacturer-provided Kunwei analysis software, with identical analysis settings applied across all trials. Consistent with the impulse–momentum framework described by Wang et al. [24], the concentric phase extended from the initiation of the upward push to hand take-off. Concentric impulse was derived from the net vertical force–time signal over this phase, and velocity was derived from the force–time signal using the impulse–momentum relationship. Instantaneous power was calculated as the product of force and velocity. Concentric peak power was defined as the highest instantaneous power during the concentric phase, whereas concentric mean power represented the average power over this phase. For each outcome, the mean value from the three formal trials was used for statistical analysis. BPU reflects upper-limb closed-chain ballistic pushing performance and should not be interpreted as a direct measure of punching speed, striking impulse, or competitive boxing performance.

Isokinetic testing was used to assess peak torque output of the self-reported dominant shoulder and knee. Before each test, the seat position, dynamometer axis, lever-arm position, and fixation devices were individually adjusted according to each participant’s anthropometry and the manufacturer’s procedures. Straps were used to stabilize the trunk and non-tested body segments to minimize compensatory movement. The dynamometer was calibrated in accordance with the manufacturer’s instructions before testing. An angular velocity of 60°·s^−1^ was selected as a standardized relatively slow testing velocity to emphasize maximal concentric torque production and test reproducibility rather than movement-speed specificity. This velocity was not intended to reproduce the angular velocity of a boxing punch. Knee testing used a concentric/concentric flexion–extension mode at an angular velocity of 60°·s^−1^ through a 90° range of motion. Participants completed 3–5 familiarization repetitions before performing five maximal consecutive knee-extension and knee-flexion actions. Shoulder testing used a concentric/concentric flexion–extension mode at 60°·s^−1^ through a 0–180° range of motion. Participants completed three familiarization repetitions, rested for 1 min, and then performed five maximal consecutive flexion–extension actions. For each movement direction, the mean of the PT values obtained across the five maximal repetitions was used for statistical analysis [27].

### 2.6. Data processing and statistical analysis

All analyzes were performed in R (version 4.4.2) [28]. Data are presented as mean ± SD, with statistical significance set at *P* < 0.05. Ten planned performance outcomes were analyzed: CMJ jump height, peak power, and relative peak power; BPU concentric impulse, concentric mean power, and concentric peak power; shoulder flexion and extension PT; and knee flexion and extension PT. No single outcome was prospectively designated as the primary endpoint; therefore, the condition × time interaction was treated as the principal inferential test for each outcome. Two-way repeated-measures ANOVA included condition (PLA, CAF3, CAF6) and time (baseline, 30 min, 60 min) as within-subject factors. Period, sequence, and carryover effects were not formally tested; counterbalancing and the 1-week washout were used only to reduce these potential influences. Baseline was included as a level of the time factor, and pre-ingestion between-condition differences were not interpreted as treatment effects. Treatment responses were interpreted primarily from the condition × time interaction.

To control family-wise error across the planned family of 10 interaction tests, *P* values were Holm-adjusted. This inferential hierarchy was not established through prospective trial registration or a publicly available statistical analysis plan. Post hoc comparisons were conducted only for outcomes with a significant Holm-adjusted interaction and were Bonferroni-adjusted separately within each outcome-specific family; no global adjustment was applied across all pairwise comparisons. Pairwise mean differences are reported with 95% CIs. For key caffeine-versus-placebo comparisons, Cohen’s dz was calculated as the paired mean difference divided by the SD of the within-participant paired differences. Data distributions and outliers were inspected before analysis. Sphericity was assessed using Mauchly’s test, with Greenhouse–Geisser correction when violated. Effect sizes are reported as partial eta squared (ηp^2^) [29].

Supplementary baseline reliability and exploratory threshold-based classification analyzes were conducted to evaluate outcome stability and the distribution of participant-level changes. Baseline reliability was assessed using baseline data from the three experimental conditions and was quantified using ICC(2,1), typical error (TE), coefficient of variation based on TE (TE CV), and mean individual coefficient of variation. Placebo-adjusted change was calculated as the change from baseline in a caffeine condition minus the corresponding change from baseline in the PLA condition at the same time point. The smallest worthwhile change (SWC) was defined as 0.2 × the PLA baseline SD, and max(SWC, TE) was used as the strict threshold. Changes exceeding the positive threshold were classified as above threshold, changes below the negative threshold were classified as below threshold, and all remaining values were classified as within threshold. Because each supplementation condition was administered only once, these classifications were considered exploratory and were not interpreted as evidence of reproducible individual responsiveness. These analyzes were descriptive and exploratory and were not part of the principal randomized group-level inferential framework. Data-completeness checks are provided in Supplementary Table S12; complete descriptive statistics and change-from-baseline summaries are provided in Supplementary Tables S3 and S4; and full reliability results are provided in Supplementary Table S5.

## 3. Results

### 3.1. Test reliability and within-participant variability

Baseline stability showed good reliability for the three CMJ outcomes (ICC(2,1) = 0.813–0.865; TE CV = 3.9%–5.4%), BPU concentric peak power (ICC(2,1) = 0.870; TE CV = 4.6%), shoulder flexion PT (ICC(2,1) = 0.800; TE CV = 11.5%), and knee flexion PT (ICC(2,1) = 0.838; TE CV = 8.9%). Moderate reliability was observed for BPU concentric impulse (ICC(2,1) = 0.564; TE CV = 13.6%), BPU concentric mean power (ICC(2,1) = 0.671; TE CV = 11.6%), shoulder extension PT (ICC(2,1) = 0.677; TE CV = 11.2%), and knee extension PT (ICC(2,1) = 0.678; TE CV = 14.8%). The corresponding typical errors for the three BPU outcomes were 11.74 N·s, 104.20 W, and 45.01 W, respectively. Shoulder extension PT also showed moderate reliability (ICC(2,1) = 0.677; TE CV = 11.2%). Accordingly, BPU effects should be interpreted in the context of this measurement error rather than from percentage changes alone. The complete reliability and within-participant variability statistics are provided in Supplementary Table S5. All 10 condition × time interactions remained statistically significant after Holm adjustment across the planned family of outcome-level interaction tests (adjusted *P* ≤ 0.011). Accordingly, outcome-specific post hoc comparisons were examined using separate Bonferroni adjustments within each outcome. Key caffeine-versus-placebo contrasts at the 60-min testing block, including 95% CIs and Cohen’s dz, are summarized in Table 3.

### 3.2. Participant flow and adverse effects

All 15 participants completed all three supplementation conditions and were included in the analyzes. No participant withdrew because of an adverse event. However, because adverse effects were assessed only verbally and were not systematically recorded using a standardized instrument, their incidence, type, and severity cannot be determined. Accordingly, the absence of withdrawals should not be interpreted as evidence that no adverse effects occurred.

### 3.3. Lower-limb explosive performance

CMJ results are shown in Figure 2 and Tables 2 and 3. Significant condition × time interactions and time main effects were observed for CMJ jump height, peak power, and relative peak power, whereas condition main effects were not significant. Post hoc comparisons showed that, during the scheduled 60-min assessment, all three CMJ outcomes were significantly higher in CAF3 and CAF6 than in PLA, with no significant difference between CAF3 and CAF6. During the scheduled 60-min assessment, compared with PLA, jump height was higher by 0.039 m in CAF3 (*P* = 0.003) and 0.051 m in CAF6 (*P* < 0.001), peak power was higher by 279.2 W in CAF3 (*P* = 0.002) and 348.3 W in CAF6 (*P* < 0.001), and relative peak power was higher by 3.60 W·kg^−1^ in CAF3 (*P* = 0.005) and 4.47 W·kg^−1^ in CAF6 (*P* = 0.001). During the scheduled 30-min assessment, only CAF6 peak power was significantly higher than PLA (difference = 179.9 W, *P* = 0.013). Overall, between-condition differences in CMJ performance were most evident during the scheduled 60-min assessment.

**Table 2.** Descriptive statistics and two-way repeated-measures analysis of variance for all performance outcomes.

Outcome	Unit	Condition	Baseline	30 min	60 min	Condition effect	ηp^2^	Time effect	ηp^2^	Condition × time	ηp^2^
CMJ jump height	m	Placebo	0.471 ± 0.079	0.456 ± 0.079*	0.450 ± 0.087*	F(2, 28) = 1.77, *P* = 0.189	0.112	F(1.4, 19.2) = 20.02, *P* < 0.001	0.588	F(2.3, 32.8) = 36.95, *P* < 0.001	0.725
		3 mg·kg^−1^	0.432 ± 0.080†	0.471 ± 0.082*	0.489 ± 0.081*†						
		6 mg·kg^−1^	0.440 ± 0.088†	0.478 ± 0.081*	0.501 ± 0.083*†						
CMJ peak power	W	Placebo	4402.7 ± 611.2	4241.7 ± 565.3*	4228.6 ± 524.4*	F(2, 28) = 2.20, *P* = 0.130	0.136	F(1.3, 18.3) = 31.14, *P* < 0.001	0.690	F(4, 56) = 45.88, *P* < 0.001	0.766
		3 mg·kg^−1^	4056.3 ± 590.8†	4305.6 ± 614.9*	4507.8 ± 634.2*†						
		6 mg·kg^−1^	4193.1 ± 569.7†	4421.6 ± 545.3*†	4576.9 ± 539.7*†						
CMJ relative peak power	W·kg^−1^	Placebo	59.40 ± 7.00	57.53 ± 6.62*	57.47 ± 6.33*	F(2, 28) = 1.54, *P* = 0.233	0.099	F(1.3, 18.7) = 29.08, *P* < 0.001	0.675	F(4, 56) = 32.47, *P* < 0.001	0.699
		3 mg·kg^−1^	54.80 ± 8.27†	58.13 ± 8.19*	61.07 ± 8.39*†						
		6 mg·kg^−1^	56.40 ± 7.04†	59.53 ± 6.12*	61.93 ± 7.17*†						
BPU concentric impulse	N·s	Placebo	91.0 ± 17.2	89.7 ± 23.3	88.6 ± 18.7	F(2, 28) = 0.90, *P* = 0.417	0.061	F(2, 28) = 6.36, *P* = 0.005	0.312	F(4, 56) = 4.11, *P* = 0.011	0.227
		3 mg·kg^−1^	85.7 ± 15.4	96.0 ± 13.0*	96.2 ± 19.8*						
		6 mg·kg^−1^	82.1 ± 21.4	101.8 ± 28.3*	100.8 ± 21.7*†						
BPU concentric mean power	W	Placebo	941.4 ± 174.9	926.9 ± 221.4	919.4 ± 184.5	F(2, 28) = 1.38, *P* = 0.269	0.089	F(2, 28) = 6.17, *P* = 0.006	0.306	F(4, 56) = 3.99, *P* = 0.011	0.222
		3 mg·kg^−1^	889.9 ± 161.6	996.5 ± 140.0*	1012.3 ± 211.0*						
		6 mg·kg^−1^	860.3 ± 219.2	1043.9 ± 292.9*	1056.7 ± 230.2*†						
BPU concentric peak power	W	Placebo	1500.5 ± 264.2	1460.7 ± 250.9	1494.4 ± 277.0	F(2, 28) = 2.80, *P* = 0.078	0.167	F(2, 28) = 11.62, *P* < 0.001	0.454	F(4, 56) = 5.25, *P* = 0.005	0.273
		3 mg·kg^−1^	1401.0 ± 229.0	1605.7 ± 225.8*	1636.5 ± 328.6*						
		6 mg·kg^−1^	1404.6 ± 315.1	1740.1 ± 479.1*	1715.1 ± 360.9*†						
Shoulder flexion PT	N·m	Placebo	76.3 ± 17.3	70.3 ± 17.0*	73.4 ± 15.8	F(1.3, 18.1) = 8.36, *P* = 0.006	0.374	F(1.3, 18.7) = 5.22, *P* = 0.026	0.271	F(4, 56) = 5.79, *P* = 0.003	0.293
		3 mg·kg^−1^	73.1 ± 19.3	77.6 ± 13.3†	82.4 ± 16.2*†						
		6 mg·kg^−1^	75.8 ± 20.6	85.7 ± 22.1†	89.7 ± 20.8*†						
Shoulder extension PT	N·m	Placebo	105.5 ± 16.6	106.7 ± 14.4	101.6 ± 13.7	F(2, 28) = 4.05, *P* = 0.029	0.224	F(1.4, 20.0) = 19.63, *P* < 0.001	0.584	F(2.2, 31.0) = 10.56, *P* = 0.001	0.430
		3 mg·kg^−1^	105.0 ± 23.6	113.2 ± 23.9*	115.1 ± 24.0*†						
		6 mg·kg^−1^	98.7 ± 21.1	117.6 ± 24.2*	125.4 ± 25.6*†						
Knee flexion PT	N·m	Placebo	128.4 ± 25.0	130.6 ± 21.2	127.6 ± 25.2	F(1.3, 18.7) = 3.23, *P* = 0.079	0.187	F(2, 28) = 14.55, *P* < 0.001	0.510	F(2.0, 28.0) = 7.82, *P* = 0.006	0.358
		3 mg·kg^−1^	127.2 ± 32.6	141.2 ± 23.4*	137.2 ± 28.2						
		6 mg·kg^−1^	120.2 ± 28.1†	140.1 ± 24.8*	153.4 ± 24.6*†						
Knee extension PT	N·m	Placebo	182.6 ± 46.6	183.7 ± 45.5	181.9 ± 47.2	F(2, 28) = 9.65, *P* < 0.001	0.408	F(2, 28) = 16.82, *P* < 0.001	0.546	F(2.4, 34.0) = 10.67, *P* < 0.001	0.432
		3 mg·kg^−1^	185.3 ± 52.2	206.0 ± 60.4†	197.3 ± 48.6						
		6 mg·kg^−1^	176.3 ± 51.7	207.3 ± 53.6*†	223.2 ± 54.8*†‡						

Note: Values are mean ± SD. Bonferroni-adjusted *P* < 0.05 versus baseline within condition; † versus PLA at the same time point; ‡ versus CAF3 at the same time point. Condition × time interaction P values were Holm-adjusted across the 10 planned outcomes, whereas post-hoc comparisons were Bonferroni-adjusted within each outcome. Baseline between-condition differences were not interpreted as treatment effects. CMJ, countermovement jump; BPU, ballistic push-up; PT, peak torque.

**Table 3.** Key caffeine-versus-placebo pairwise comparisons at the 60-min testing block.

Outcome (unit)	Contrast	Mean difference (95% CI)	Cohen’s dz	Bonferroni-adjusted P
CMJ jump height (m)	CAF3 − PLA	0.039 (0.019 to 0.059)	1.06	0.003
CMJ jump height (m)	CAF6 − PLA	0.051 (0.032 to 0.071)	1.44	<0.001
CMJ peak power (W)	CAF3 − PLA	279.2 (140.4 to 418.0)	1.11	0.002
CMJ peak power (W)	CAF6 − PLA	348.3 (221.4 to 475.3)	1.52	<0.001
CMJ relative peak power (W·kg^−1^)	CAF3 − PLA	3.60 (1.61 to 5.59)	1.00	0.005
CMJ relative peak power (W·kg^−1^)	CAF6 − PLA	4.47 (2.39 to 6.55)	1.19	0.001
BPU concentric impulse (N·s)	CAF6 − PLA	12.20 (3.03 to 21.37)	0.74	0.038
BPU concentric mean power (W)	CAF6 − PLA	137.3 (41.8 to 232.7)	0.80	0.024
BPU concentric peak power (W)	CAF6 − PLA	220.7 (79.7 to 361.6)	0.87	0.014
Shoulder flexion PT (N·m)	CAF3 − PLA	9.07 (4.19 to 13.94)	1.03	0.004
Shoulder flexion PT (N·m)	CAF6 − PLA	16.33 (7.30 to 25.36)	1.00	0.005
Shoulder extension PT (N·m)	CAF3 − PLA	13.44 (3.48 to 23.41)	0.75	0.035
Shoulder extension PT (N·m)	CAF6 − PLA	23.82 (14.82 to 32.82)	1.47	<0.001
Knee flexion PT (N·m)	CAF6 − PLA	25.78 (16.24 to 35.31)	1.50	<0.001
Knee extension PT (N·m)	CAF6 − PLA	41.36 (28.96 to 53.75)	1.85	<0.001

Mean differences are expressed as caffeine minus placebo; positive values therefore indicate higher performance under caffeine. The 95% CIs are conventional two-sided intervals; *P* values were Bonferroni-adjusted within the corresponding outcome-specific post-hoc family; no global adjustment was applied across pairwise comparisons from different outcomes. Cohen’s dz was calculated as the paired mean difference divided by the SD of the within-participant paired differences. PLA, placebo; CAF3, 3 mg·kg⁻¹ caffeine; CAF6, 6 mg·kg⁻¹ caffeine; CI, confidence interval.

**Figure 2.**
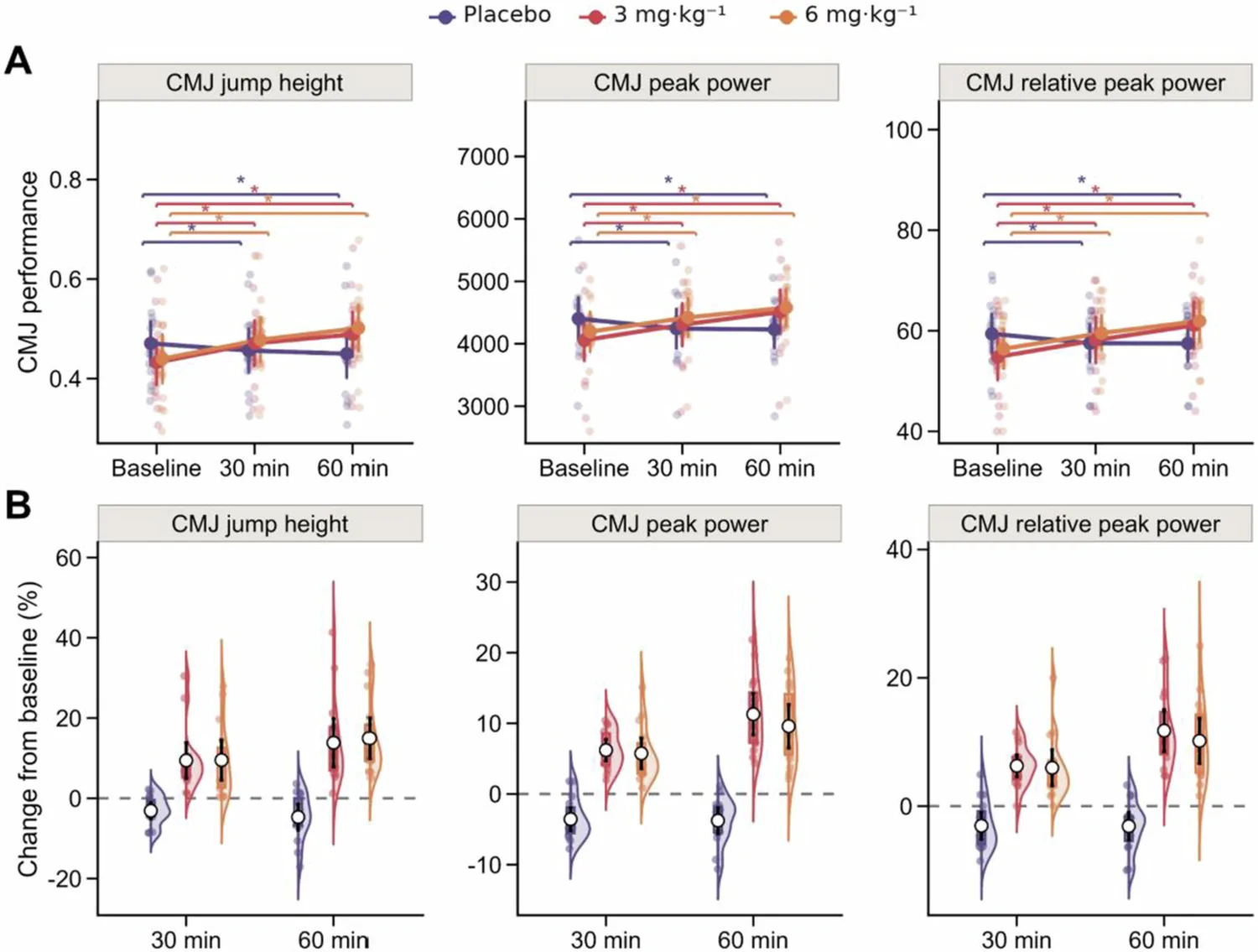
Countermovement jump performance and changes from baseline across supplementation conditions. A, absolute values for CMJ outcomes; B, changes from baseline within each condition. Outcomes include CMJ jump height, peak power, and relative peak power. Data are mean ± SD. PLA, placebo; CAF3, 3 mg·kg^−1^ caffeine; CAF6, 6 mg·kg^−1^ caffeine; CMJ, countermovement jump. *Bonferroni-adjusted *P* < 0.05 vs. baseline within the same condition; †Bonferroni-adjusted *P* < 0.05 vs. PLA at the same time point; ‡Bonferroni-adjusted *P* < 0.05 vs. CAF3 at the same time point.

### 3.4. Upper-limb explosive performance

BPU results are shown in Figure 3 and Tables 2 and 3. Significant condition × time interactions and time main effects were observed for BPU concentric impulse, concentric mean power, and concentric peak power, whereas condition main effects were not significant. Post hoc comparisons showed that, during the scheduled 60-min assessment, CAF6 produced higher concentric impulse, concentric mean power, and concentric peak power than PLA, with differences of 12.20 N·s (*P* = 0.038), 137.3 W (*P* = 0.024), and 220.7 W (*P* = 0.014), respectively. For context, these absolute differences were approximately 1.0–1.3 times the corresponding baseline typical errors, emphasizing the need to interpret the effects alongside measurement precision. No significant differences were observed between CAF3 and PLA or between CAF3 and CAF6, and no significant between-condition differences were observed during the scheduled 30-min assessment. Thus, statistically significant between-condition differences in BPU outcomes were observed only for CAF6 versus PLA during the scheduled 60-min assessment, but their practical magnitude should be interpreted cautiously in the context of measurement error.

**Figure 3.**
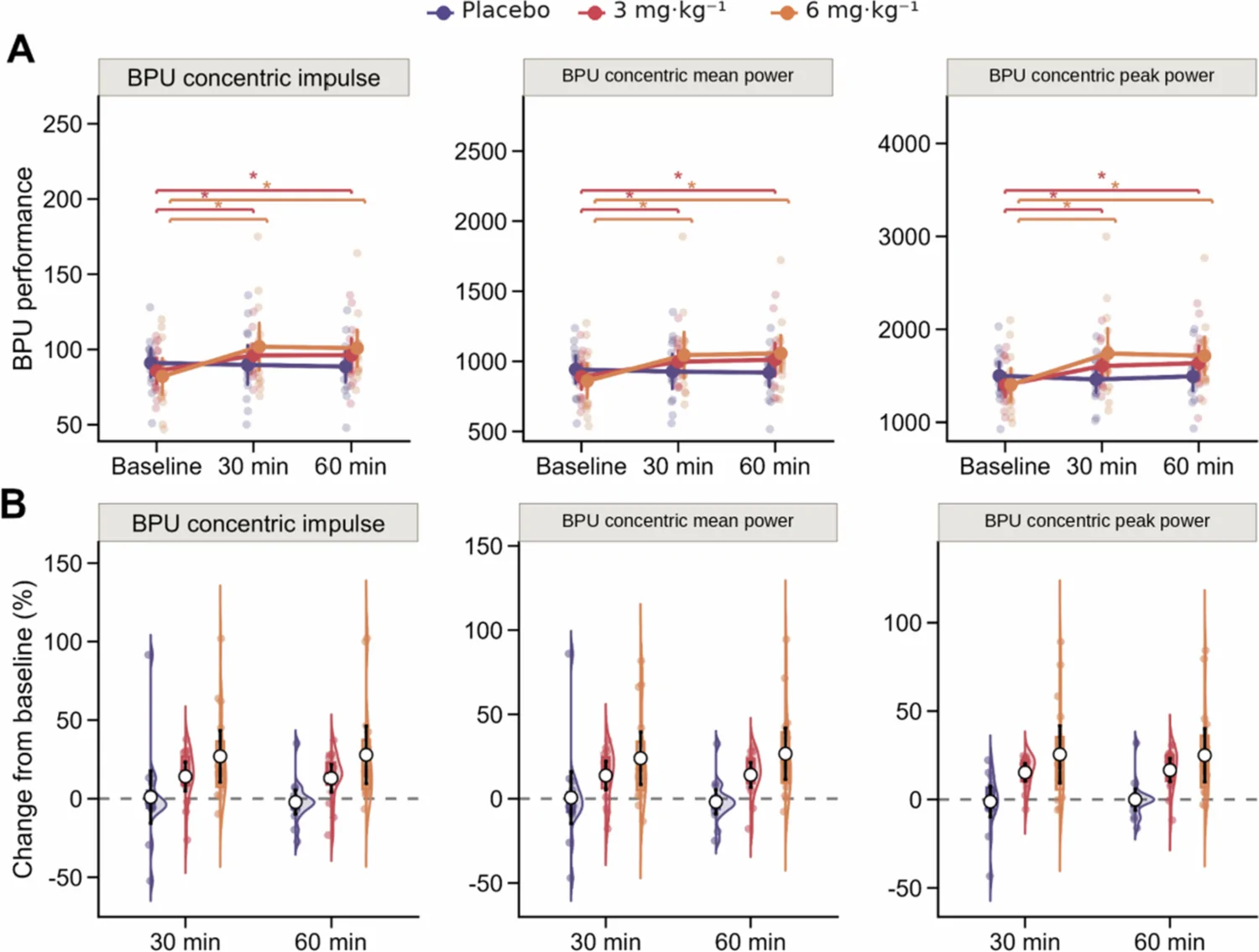
Ballistic push-up performance and changes from baseline across supplementation conditions. A, absolute values for BPU outcomes; B, changes from baseline within each condition. Outcomes include concentric impulse, concentric mean power, and concentric peak power. BPU reflects upper-limb closed-chain ballistic pushing performance rather than direct boxing punching performance. Data are mean ± SD. PLA, placebo; CAF3, 3 mg·kg^−1^ caffeine; CAF6, 6 mg·kg^−1^ caffeine; BPU, ballistic push-up. *Bonferroni-adjusted *P* < 0.05 vs. baseline within the same condition; †Bonferroni-adjusted *P* < 0.05 vs. PLA at the same time point; ‡Bonferroni-adjusted *P* < 0.05 vs. CAF3 at the same time point.

### 3.5. Shoulder isokinetic peak torque

Shoulder isokinetic peak torque results are shown in Figure 4 and Tables 2 and 3. Significant condition × time interactions, time main effects, and condition main effects were observed for shoulder flexion and extension PT. Shoulder flexion PT was significantly higher than baseline during the scheduled 60-min assessment after CAF3 and CAF6 ingestion. Compared with PLA, both caffeine conditions produced higher shoulder flexion PT during the scheduled 30- and 60-min assessments.

Shoulder extension PT was significantly higher than baseline during both the scheduled 30- and 60-min assessments after CAF3 and CAF6 ingestion, and CAF6 during the scheduled 60-min assessment was significantly higher than its value during the scheduled 30-min assessment (difference = 7.87 N·m, *P* = 0.022). Between-condition comparisons showed that CAF3 and CAF6 were higher than PLA during the scheduled 60-min assessment by 13.44 N·m (*P* = 0.035) and 23.82 N·m (*P* < 0.001), respectively. The difference between CAF6 and CAF3 did not reach statistical significance (*P* = 0.064). Overall, caffeine improved selected shoulder PT outcomes, but no clear dose advantage was observed.

### 3.6. Knee isokinetic peak torque

Knee isokinetic peak torque results are shown in Figure 4 and Tables 2 and 3. For knee flexion PT, the condition main effect was not significant, whereas the time main effect and condition × time interaction were significant. CAF3 was significantly higher than baseline only during the scheduled 30-min assessment. CAF6 was significantly higher than baseline during both the scheduled 30- and 60-min assessments, and the value during the scheduled 60-min assessment was higher than that during the scheduled 30-min assessment. Between-condition comparisons showed that CAF6 was significantly higher than PLA during the scheduled 60-min assessment, whereas the difference between CAF6 and CAF3 did not reach statistical significance.

For knee extension PT, the condition main effect, time main effect, and condition × time interaction were all significant. CAF6 was significantly higher than baseline during both the scheduled 30- and 60-min assessments, and the value during the scheduled 60-min assessment was higher than that during the scheduled 30-min assessment (difference = 15.91 N·m, *P* = 0.003). Between-condition comparisons showed that CAF3 and CAF6 were both significantly higher than PLA during the scheduled 30-min assessment. During the scheduled 60-min assessment, CAF6 was significantly higher than PLA (difference = 41.36 N·m, *P* < 0.001) and CAF3 (difference = 25.98 N·m, *P* = 0.021). Therefore, the clearest CAF6 effect on knee extension PT occurred during the scheduled 60-min assessment.

**Figure 4.**
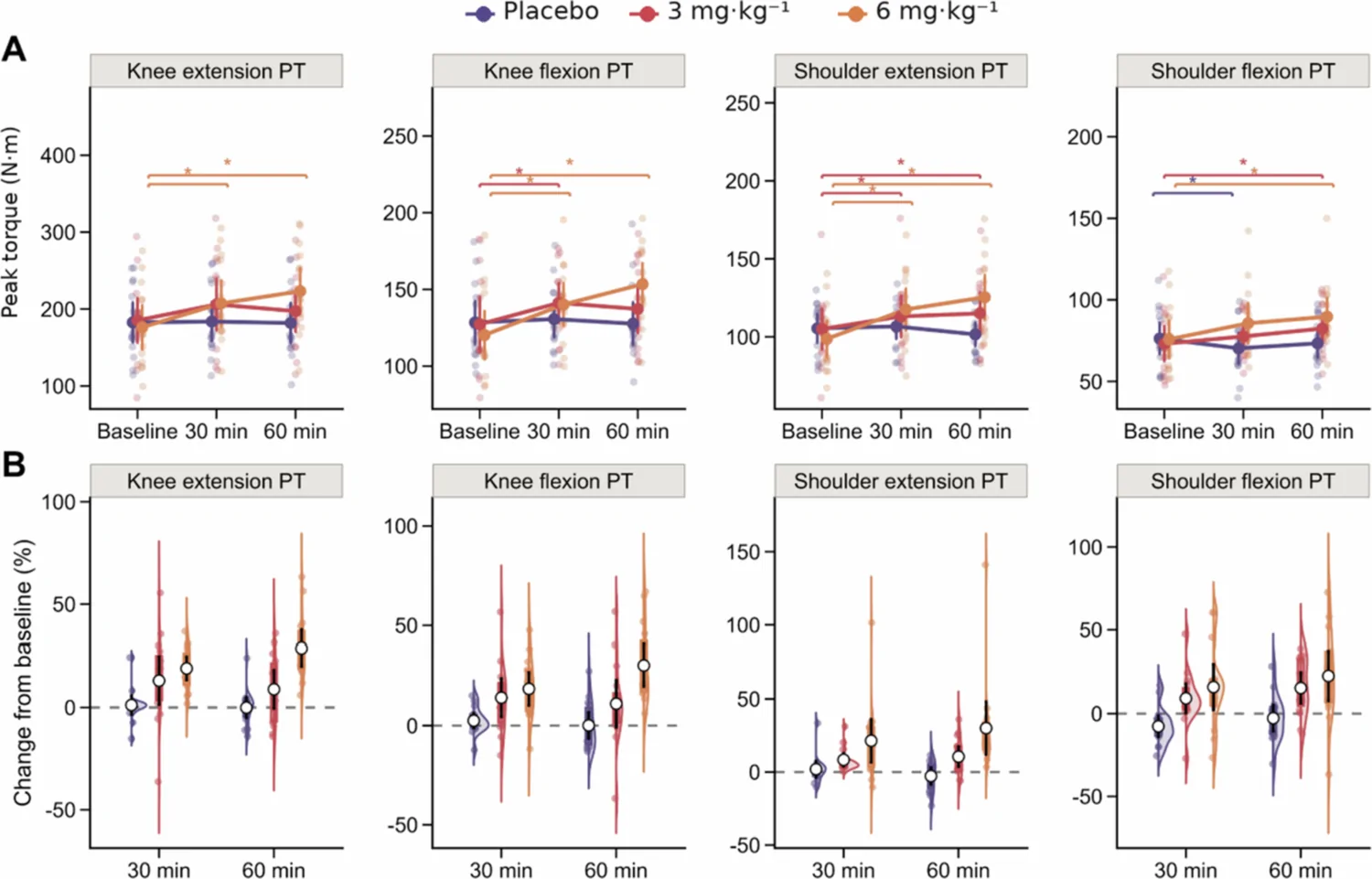
Shoulder and knee isokinetic peak torque performance and changes from baseline across supplementation conditions. A, absolute isokinetic PT values for the shoulder and knee; B, changes from baseline within each condition. Outcomes include knee extension PT, knee flexion PT, shoulder extension PT, and shoulder flexion PT. Data are mean ± SD. PLA, placebo; CAF3, 3 mg·kg^−1^ caffeine; CAF6, 6 mg·kg^−1^ caffeine; PT, peak torque. *Bonferroni-adjusted *P* < 0.05 vs. baseline within the same condition; †Bonferroni-adjusted *P* < 0.05 vs. PLA at the same time point; ‡Bonferroni-adjusted *P* < 0.05 vs. CAF3 at the same time point.

### 3.7. Exploratory individual-change analyzes

Exploratory threshold-based analyzes were conducted to describe the distribution of placebo-adjusted individual changes. Because each caffeine dose was administered only once, these classifications were not interpreted as evidence of reproducible individual responsiveness or used to identify “true responders.” Detailed results are provided in Supplementary Tables S6a–S10 and Supplementary Figures S1–S3.

## 4. Discussion

### 4.1. Main findings

The main finding of this randomized, double-blind, placebo-controlled crossover trial was that response patterns varied according to caffeine dose, post-ingestion timing, and testing task. The clearest between-condition differences were observed during the scheduled 60-min assessment. CMJ outcomes were higher after both CAF3 and CAF6 than after PLA during the scheduled 60-min assessment, whereas statistically significant between-condition differences in BPU outcomes were observed only for CAF6 versus PLA. For isokinetic peak torque, an outcome-specific between-dose difference was observed for knee extension PT, with CAF6 exceeding both PLA and CAF3 during the scheduled 60-min assessment. Thus, caffeine was not associated with uniform changes across all strength–power outcomes.

The boxing context of these findings also requires careful interpretation. Lower-limb force and power contribute to stance control, rapid repositioning, and the initiation of whole-body force transmission during punching, whereas upper-body force expression represents only the terminal component of a more complex kinetic-chain action. Accordingly, the CMJ, BPU, and isokinetic PT tests capture different physical qualities that may contribute to boxing performance, but none reproduces the coordination, rotational mechanics, timing, or impact characteristics of an actual punch. The present findings should therefore be viewed as evidence of caffeine-related changes in selected physical capacities relevant to boxing rather than direct improvements in boxing-specific performance.

### 4.2. Dose-, time-, and task-specific responses

The different patterns observed for CMJ, BPU, and isokinetic PT suggest that the acute effect of caffeine depends partly on the task used to express neuromuscular performance. CMJ showed the most consistent pattern: both caffeine doses produced higher jump height and power than PLA during the scheduled 60-min assessment, with no detectable difference between CAF3 and CAF6. By contrast, statistically significant between-condition differences in BPU outcomes were observed only for CAF6 versus PLA during the scheduled 60-min assessment. For context, the CAF6–PLA differences in concentric impulse and concentric mean power were approximately 1.0 and 1.3 times their corresponding baseline typical errors, respectively, whereas the concentric peak-power difference was substantially larger than its typical error. The BPU findings should nevertheless be interpreted alongside outcome-specific measurement precision. Isokinetic PT responses were also non-uniform. Shoulder flexion and extension PT were higher under caffeine conditions at selected time points but without a consistent dose advantage, whereas knee extension PT showed the clearest CAF6 response during the scheduled 60-min assessment. This pattern supports a dose-, time-, and task-dependent interpretation rather than a generalized caffeine benefit across all outcomes.

The present findings also suggest that caffeine responses were not uniform across upper- and lower-body tasks. CMJ outcomes improved after both CAF3 and CAF6, whereas statistically significant between-condition differences in BPU outcomes were observed only for CAF6 versus PLA. However, this pattern should not be interpreted as evidence that lower-body musculature is inherently more responsive to caffeine. CMJ and BPU differed in movement complexity, coordination demands, and measurement reliability, with greater variability observed for BPU outcomes. Therefore, the present data support a task-dependent rather than a purely upper- versus lower-body interpretation of caffeine effects [9,14,17]. The dose-response pattern was outcome dependent. Both CAF3 and CAF6 improved CMJ outcomes during the scheduled 60-min assessment, with no significant difference between doses, whereas knee-extension PT was significantly higher with CAF6 than with CAF3 at this assessment. This finding should be interpreted as an outcome-specific between-dose difference rather than evidence of a generalized dose-response relationship. This pattern is broadly consistent with Pallarés et al. [18], who reported that increasing caffeine dose did not uniformly translate into greater neuromuscular performance benefits and that higher doses were accompanied by a greater incidence of side effects. The ISSN position stand by Guest et al. [7] similarly identifies 3–6 mg·kg^−1^ as a commonly effective range for exercise performance, without implying that the upper end of this range is necessarily superior. In addition, the meta-analysis by Grgic et al. [9] supports an ergogenic effect of caffeine on strength and power outcomes, providing a broader context for the present findings. Taken together, the current results and previous evidence suggest that caffeine responses are task- and outcome-dependent, and do not support a uniform performance advantage of 6 mg·kg^−1^ over 3 mg·kg^−1^.

The more consistent effects observed during the scheduled 60-min assessment may partly reflect the absorption profile of encapsulated caffeine. Plasma caffeine concentrations rise after ingestion, but the time to peak concentration varies considerably and may extend beyond 60 min [11]. Previous work has also shown that the timing of caffeine ingestion can influence strength–power responses, with different outcomes responding at earlier or later post-ingestion assessments [12]. In addition, genetic variation related to caffeine metabolism, particularly CYP1A2 polymorphisms, may contribute to interindividual variability in caffeine responses [13]. However, plasma or salivary caffeine concentrations and genotype were not assessed in the present study. Therefore, the scheduled 60-min assessment should not be interpreted as corresponding to a pharmacokinetic peak. Differences between the scheduled 30- and 60-min assessments should instead be interpreted as time-specific performance responses rather than evidence of individual pharmacokinetic or genetic effects. The within-block timing of the assessments should also be considered when interpreting these time-dependent responses. Because tests were performed sequentially within each ~15-min block, the shoulder and knee assessments occurred later after ingestion than the preceding force-platform tests. Thus, apparent differences in the time course across tasks may partly reflect these timing offsets rather than task-specific caffeine responses alone. Several mechanisms have been proposed in the caffeine literature, including adenosine-receptor antagonism, altered arousal and perceived effort, changes in voluntary neural drive or motor-unit behavior, and peripheral effects on excitation–contraction processes [7,8,30–33]. However, none of these mechanisms was directly assessed in the present study. Plasma or salivary caffeine concentrations, adenosine signaling, neural drive, motor-unit recruitment, and excitation–contraction coupling were not measured. Accordingly, these mechanisms should be considered only as possible explanations from the existing literature and cannot be inferred from the present performance outcomes.

Task characteristics may help explain why responses were not uniform. CMJ is a standardized bilateral lower-limb explosive task that primarily reflects lower-limb extension power and vertical force-velocity output [34]. In this study, CMJ outcomes showed good baseline reliability and relatively low measurement error, making caffeine-related changes easier to detect. BPU validly assesses upper-limb closed-chain pushing power [24–26], but it also depends on shoulder-girdle stability, elbow-extension strategy, hand placement, trunk control, and push-off technique. Within-participant variability was larger for BPU concentric mean power and concentric impulse than for CMJ outcomes. Any contribution of altered neural drive or perceived effort cannot be determined from the present data; technical and coordination variability may also have influenced the observed BPU responses.

The present CMJ findings are consistent with meta-analytic evidence that caffeine can produce small beneficial effects on muscular strength and power [9] and with work showing improved vertical jump performance after caffeine ingestion in collegiate athletes [35]. However, other studies have reported null effects on squat jump or CMJ height [16]. Training background, habitual caffeine intake, sport characteristics, dosing strategy, and test reliability may all moderate caffeine responses. The BPU findings should be interpreted even more cautiously. Although statistically significant CAF6–PLA differences were observed during the scheduled 60-min assessment, the condition main effect was not significant and some BPU outcomes showed relatively large measurement variability. Accordingly, the relatively large percentage changes observed for some BPU outcomes should not be interpreted in isolation; the absolute between-condition differences, confidence intervals, standardized effect sizes, and measurement-error estimates provide a more appropriate basis for judging their magnitude. Moreover, BPU reflects closed-chain push-off ability under fixed support conditions, whereas boxing punches are open-chain, rotational, whole-body actions involving lower-limb drive, trunk rotation, shoulder-elbow-wrist acceleration, and striking timing [2,3]. Therefore, BPU improvements should not be equated with improved punching speed, striking impulse, or competitive attacking quality.

### 4.3. Isokinetic peak torque responses

The isokinetic peak torque results further support a task-specific interpretation. Shoulder flexion and extension PT improved at selected time points after CAF3 and CAF6, but differences between doses were not stable. For the knee, CAF6 produced the clearest effect, particularly for knee extension PT during the scheduled 60-min assessment. Isokinetic testing assesses maximal voluntary joint torque under a fixed angular velocity and range of motion. It is less influenced by whole-body movement strategy than multi-joint explosive tasks such as CMJ and BPU. Accordingly, isokinetic PT provides complementary information on whether caffeine affects torque output in specific joints or muscle groups.

Previous meta-analyzes suggest that caffeine can improve muscular strength and isokinetic strength, with relatively clear responses in knee extensors [36,37]. One study reported that 6 mg·kg^−1^ caffeine improved knee-extensor strength, power, and endurance across different angular velocities in resistance-trained men, whereas knee-flexor responses were more limited [38]. These findings are consistent with the clear CAF6 pattern for knee extension PT during the scheduled 60-min assessment in the present study. However, this pattern should not be generalized beyond the tested participants, dose conditions, and measurement protocol.

The prominent knee-extension response may be related to the high torque-producing capacity of the quadriceps [39] and to the role of lower-limb extension in stance support, footwork, and kinetic-chain force transmission during boxing [2,3,40]. One possibility is that the standardized knee-extension task provided a relatively stable context in which caffeine-related changes in maximal voluntary effort could be expressed. However, no measures of central excitability, neural drive, or motor-unit behavior were collected, so this explanation remains speculative. By contrast, although knee flexion PT increased under CAF6, the between-dose difference did not reach statistical significance. This response should therefore be interpreted as less stable. Previous isokinetic studies often evaluate hamstring-to-quadriceps PT ratios and indicate that knee flexor torque is generally lower than knee extensor torque [41]. Isokinetic knee testing may also be affected by stabilization, trunk control, movement control, and test familiarization [42]. Weak or borderline isokinetic differences should therefore not be inferred solely from numerical direction.

Shoulder PT improved under caffeine conditions but did not show a stable dose advantage. Shoulder testing requires substantial joint stability, movement control, and familiarization, and caffeine effects may be influenced by muscle-group size, coordination demands, and measurement error. Similarly, percentage changes in isokinetic PT should be interpreted alongside the absolute N·m differences and measurement-error estimates, particularly for shoulder extension PT, which showed only moderate reliability. Prior work has shown that moderate-dose caffeine can enhance maximal voluntary contraction strength in several upper- and lower-body muscle groups in resistance-trained men, but whether muscle location and size moderate this effect remains uncertain [43]. From a boxing perspective, the relevance of these laboratory-based outcomes is supported by previous research. Loturco et al. [3] reported strong associations between strength–power qualities and punching impact in elite amateur boxers, while Chaabène et al. [1] described the broader physical and physiological demands of amateur boxing. Isokinetic knee and shoulder strength have also been associated with punching force or boxing-specific strength performance [4,5]. These findings provide a rationale for considering CMJ, BPU, and isokinetic PT as boxing-relevant neuromuscular measures. However, such associations do not indicate that caffeine-induced improvements in these laboratory outcomes translate directly into greater punching performance. Punching is a whole-body kinetic-chain skill involving lower-limb force production, trunk rotation, upper-limb acceleration, timing, and technical execution [2,3]. Therefore, the present findings should be interpreted as changes in selected neuromuscular capacities relevant to boxing rather than direct evidence of improved punching or competitive performance.

### 4.4. Limitations

Several limitations should be acknowledged. First, the modest sample size limits the precision of between-dose estimates and precludes interpreting nonsignificant CAF3–CAF6 comparisons as evidence of equivalence. Second, although supplementation order was counterbalanced and conditions were separated by a 1-week washout, period, sequence, and carryover effects were not formally assessed. Third, plasma or salivary caffeine concentrations, caffeine-metabolism genotype, habitual caffeine intake, caffeine sensitivity, sleep quality, and pre-trial dietary intake were not systematically assessed. Meal timing and composition were not standardized, and the 48-h caffeine-abstinence period may have induced withdrawal-related effects. Adverse effects were assessed only verbally, and blinding success was not formally evaluated. Fourth, no dedicated strength-endurance assessment was included. Fifth, exact outcome-specific testing times were not prospectively recorded, and force-platform analyzes were limited to selected global performance and mechanical-output variables. Sixth, CMJ, BPU, and isokinetic PT were laboratory-based measures and did not directly assess punching or competitive boxing performance. Finally, the trial was not prospectively registered, the full protocol and statistical analysis plan were not publicly available in advance, and no single primary outcome was prospectively designated, which limits transparency regarding the prospective specification of outcomes and analyzes. Accordingly, the findings should be interpreted as changes in boxing-relevant neuromuscular capacities rather than direct improvements in boxing performance.

## 5. Conclusions

Within the conditions of this study, both 3 and 6 mg·kg^−1^ caffeine were associated with higher CMJ values than placebo during the scheduled 60-min assessment. An outcome-specific between-dose difference was observed for knee extension PT during the scheduled 60-min assessment, whereas 6 mg·kg^−1^ caffeine was not consistently superior to 3 mg·kg^−1^ across other outcomes. Overall, the response patterns varied according to caffeine dose, post-ingestion timing, and testing task. Because boxing-specific performance and standardized adverse-effect outcomes were not assessed, these findings do not establish an optimal caffeine dose or demonstrate improvements in punching or competitive performance. Further research should examine larger and more diverse athlete populations, incorporate physiological and behavioral factors relevant to caffeine responsiveness, evaluate additional post-ingestion time points, and include boxing-specific performance outcomes to determine whether laboratory-based neuromuscular changes translate into meaningful sport-specific benefits.

## 6. Practical applications

Caffeine doses of 3–6 mg·kg^−1^ may be considered when targeting selected laboratory-based strength–power outcomes in trained boxers. However, 6 mg·kg^−1^ was not consistently superior to 3 mg·kg^−1^, and the BPU findings should be interpreted cautiously in light of measurement error. Because habitual caffeine intake, potential withdrawal effects, meal standardization, and blinding were not fully controlled, the present findings do not establish an optimal competition dose applicable to all boxers. As boxing-specific performance was not assessed, these results should not be interpreted as evidence of improved punching or competitive performance.

## Supplementary Material

Supplementary Material Supplementary_Material_R3_.docx

## Data Availability

The data supporting the findings of this study are available from the corresponding author upon reasonable request.

## Protocol availability

The full study protocol and statistical analysis plan were not publicly registered or posted.

## Trial registration

This trial was not prospectively registered.
