# Supplementary material for: Acute effects of 3 and 6 mg·Kg−1 caffeine on strength–power performance and isokinetic peak torque in competitive male boxers: a randomized, double-blind, placebo-controlled crossover trial
Source: J Int Soc Sports Nutr. 2026 Oct 7;23(1):2743368. doi: 10.1080/15502783.2026.2743368 (PMC13647396; doi:10.1080/15502783.2026.2743368)
Supplement: Supplementary file 1 [file RSSN_A_2743368_SM3739.docx]

**Supplementary Material**

**Acute Effects of 3 and 6 mg·kg⁻¹ Caffeine on Strength–Power Performance and Isokinetic Peak Torque in Competitive Male Boxers: A Randomized, Double-Blind, Placebo-Controlled Crossover Trial**

This supplementary file provides additional methodological details, data-completeness checks, descriptive statistics, change scores, reliability results, and individual-response summaries for the trial.

**Abbreviations:** PLA, placebo; CAF3, 3 mg·kg⁻¹ caffeine; CAF6, 6 mg·kg⁻¹ caffeine; CMJ, countermovement jump; BPU, ballistic push-up; PT, peak torque; SWC, smallest worthwhile change; TE, typical error; CV, coefficient of variation.

Formatting note: In long supplementary tables, repeated outcome, domain, unit, and threshold entries are displayed only once within each outcome block to improve readability; blank cells indicate the same value as the first row of that block.

# Contents

| **Item** | **Page** |
| --- | --- |
| Supplementary Methods | 2 |
| Supplementary Table S1. Detailed testing protocols and scoring rules | 2 |
| Supplementary Table S2. Balanced Latin-square supplementation sequences | 3 |
| Supplementary Table S3. Complete descriptive statistics | 4 |
| Supplementary Table S4. Change from baseline | 5 |
| Supplementary Table S5. Baseline reliability and within-participant variability | 7 |
| Supplementary Table S6a. Strict individual-response thresholds and responder summary for CMJ and BPU outcomes | 8 |
| Supplementary Table S6b. Strict individual-response thresholds and responder summary for isokinetic peak torque outcomes | 9 |
| Supplementary Table S7. Domain-level responder summary using the strict threshold | 10 |
| Supplementary Table S8. Co-response across CAF3 and CAF6 at the same time point | 11 |
| Supplementary Table S9. Co-response across 30 and 60 min within the same caffeine dose | 12 |
| Supplementary Table S10. Subject-level response profile across all outcomes using the strict threshold | 13 |
| Supplementary Table S11. Key Bonferroni-adjusted pairwise comparisons | 15 |
| Supplementary Figures | 17 |
| Supplementary Figure S1. Individual placebo-adjusted responses based on the strict SWC/TE threshold | 17 |
| Supplementary Figure S2. Responder rates for placebo-adjusted changes | 18 |
| Supplementary Figure S3. Subject-level responder profile across all outcomes | 19 |

# Supplementary Methods

**Exploratory individual-response classification.** For each outcome, placebo-adjusted change was calculated as the change from baseline in a caffeine condition minus the corresponding change from baseline in the PLA condition at the same time point. The SWC was defined as 0.2 × the PLA baseline SD. To avoid classifying changes smaller than measurement error as meaningful, the strict response threshold was defined as max(SWC, TE). A positive response was classified when the placebo-adjusted change exceeded this threshold; a negative response was classified when it was below the negative threshold; all other values were classified as unclear/trivial.

Supplementary Table S1. Detailed testing protocols and scoring rules.

| **Test** | **Construct assessed** | **Protocol summary** | **Familiarization/formal trials** | **Recovery** | **Analysis variable** |
| --- | --- | --- | --- | --- | --- |
| CMJ | Lower-limb vertical explosive performance | Hands on hips; rapid countermovement to approximately 90 degrees knee flexion followed by maximal vertical jump | 5 CMJs: first 2 for warm-up/movement preparation; last 3 formal trials | 1 min between formal trials | Mean jump height, peak power, and relative peak power from the 3 formal trials |
| BPU | Upper-limb closed-chain ballistic pushing performance | Standard push-up position; hand spacing matched to bench-press grip; lower until upper arms are approximately parallel to floor; push off force platform maximally | 2-3 practice trials, then 3 formal trials | 1 min between formal trials | Concentric impulse, concentric mean power, and concentric peak power averaged across the 3 formal trials. |
| Knee isokinetic PT | Dominant-side knee maximal torque | ISOMED 2000; concentric/concentric flexion-extension; 60 degrees/s; 90 degrees range of motion; trunk and non-tested segments stabilized | 3-5 familiarization repetitions; 5 maximal repetitions | Standardized rest according to protocol | Mean peak torque from 5 repetitions |
| Shoulder isokinetic PT | Dominant-side shoulder maximal torque | ISOMED 2000; concentric/concentric flexion-extension; 60 degrees/s; 0-180 degrees range of motion; seated position with trunk stabilization | 3 familiarization repetitions; 5 maximal repetitions | 1 min after familiarization before formal test | Mean peak torque from 5 repetitions |

*CMJ and BPU were performed using a portable force platform; isokinetic testing was performed using ISOMED 2000. All testing procedures were performed at baseline and repeated during testing blocks beginning at 30 and 60 min post-ingestion in the same order.*

Supplementary Table S2. Balanced Latin-square supplementation sequences.

| **Sequence** | **Trial order** | **Participants (n)** |
| --- | --- | --- |
| Sequence A | PLA -> CAF3 -> CAF6 | 5 |
| Sequence B | CAF3 -> CAF6 -> PLA | 5 |
| Sequence C | CAF6 -> PLA -> CAF3 | 5 |

*The table summarizes the planned balanced sequence structure. Each supplementation condition appeared equally often in each formal trial position.*

Supplementary Table S3. Complete descriptive statistics.

| **Outcome** | **Domain** | **Unit** | **Condition** | **Baseline** | **30 min** | **60 min** |
| --- | --- | --- | --- | --- | --- | --- |
| CMJ jump height | Lower-limb power | m | Placebo | 0.471 ± 0.079 | 0.456 ± 0.079 | 0.450 ± 0.087 |
|  |  |  | 3 mg·kg⁻¹ | 0.432 ± 0.080 | 0.471 ± 0.082 | 0.489 ± 0.081 |
|  |  |  | 6 mg·kg⁻¹ | 0.440 ± 0.088 | 0.478 ± 0.081 | 0.501 ± 0.083 |
| CMJ peak power | Lower-limb power | W | Placebo | 4402.7 ± 611.2 | 4241.7 ± 565.3 | 4228.6 ± 524.4 |
|  |  |  | 3 mg·kg⁻¹ | 4056.3 ± 590.8 | 4305.6 ± 614.9 | 4507.8 ± 634.2 |
|  |  |  | 6 mg·kg⁻¹ | 4193.1 ± 569.7 | 4421.6 ± 545.3 | 4576.9 ± 539.7 |
| CMJ relative peak power | Lower-limb power | W·kg⁻¹ | Placebo | 59.40 ± 7.00 | 57.53 ± 6.62 | 57.47 ± 6.33 |
|  |  |  | 3 mg·kg⁻¹ | 54.80 ± 8.27 | 58.13 ± 8.19 | 61.07 ± 8.39 |
|  |  |  | 6 mg·kg⁻¹ | 56.40 ± 7.04 | 59.53 ± 6.12 | 61.93 ± 7.17 |
| BPU concentric impulse | Upper-limb power | N·s | Placebo | 91.00 ± 17.20 | 89.73 ± 23.30 | 88.60 ± 18.71 |
|  |  |  | 3 mg·kg⁻¹ | 85.67 ± 15.37 | 96.00 ± 13.02 | 96.20 ± 19.79 |
|  |  |  | 6 mg·kg⁻¹ | 82.13 ± 21.43 | 101.80 ± 28.31 | 100.80 ± 21.67 |
| BPU concentric mean power | Upper-limb power | W | Placebo | 941.4 ± 174.9 | 926.9 ± 221.4 | 919.4 ± 184.5 |
|  |  |  | 3 mg·kg⁻¹ | 889.9 ± 161.6 | 996.5 ± 140.0 | 1012.3 ± 211.0 |
|  |  |  | 6 mg·kg⁻¹ | 860.3 ± 219.2 | 1043.9 ± 292.9 | 1056.7 ± 230.2 |
| BPU concentric peak power | Upper-limb power | W | Placebo | 1500.5 ± 264.2 | 1460.7 ± 250.9 | 1494.4 ± 277.0 |
|  |  |  | 3 mg·kg⁻¹ | 1401.0 ± 229.0 | 1605.7 ± 225.8 | 1636.5 ± 328.6 |
|  |  |  | 6 mg·kg⁻¹ | 1404.6 ± 315.1 | 1740.1 ± 479.1 | 1715.1 ± 360.9 |
| Shoulder flexion PT | Isokinetic peak torque | N·m | Placebo | 76.31 ± 17.25 | 70.31 ± 17.01 | 73.36 ± 15.82 |
|  |  |  | 3 mg·kg⁻¹ | 73.09 ± 19.27 | 77.60 ± 13.29 | 82.42 ± 16.16 |
|  |  |  | 6 mg·kg⁻¹ | 75.82 ± 20.63 | 85.67 ± 22.09 | 89.69 ± 20.79 |
| Shoulder extension PT | Isokinetic peak torque | N·m | Placebo | 105.47 ± 16.56 | 106.71 ± 14.35 | 101.62 ± 13.68 |
|  |  |  | 3 mg·kg⁻¹ | 105.04 ± 23.61 | 113.16 ± 23.93 | 115.07 ± 24.02 |
|  |  |  | 6 mg·kg⁻¹ | 98.73 ± 21.10 | 117.58 ± 24.17 | 125.44 ± 25.60 |
| Knee flexion PT | Isokinetic peak torque | N·m | Placebo | 128.40 ± 25.03 | 130.62 ± 21.20 | 127.64 ± 25.16 |
|  |  |  | 3 mg·kg⁻¹ | 127.22 ± 32.57 | 141.20 ± 23.39 | 137.20 ± 28.19 |
|  |  |  | 6 mg·kg⁻¹ | 120.22 ± 28.14 | 140.11 ± 24.83 | 153.42 ± 24.57 |
| Knee extension PT | Isokinetic peak torque | N·m | Placebo | 182.60 ± 46.64 | 183.73 ± 45.52 | 181.89 ± 47.16 |
|  |  |  | 3 mg·kg⁻¹ | 185.27 ± 52.16 | 206.04 ± 60.40 | 197.27 ± 48.60 |
|  |  |  | 6 mg·kg⁻¹ | 176.33 ± 51.69 | 207.33 ± 53.64 | 223.24 ± 54.81 |

*Values are mean ± SD. All outcomes were assessed at baseline, 30 min, and 60 min under each supplementation condition.*

Supplementary Table S4. Change from baseline.

| **Outcome** | **Domain** | **Unit** | **Condition** | **Time** | **Absolute change, mean ± SD** | **Percent change, mean ± SD** |
| --- | --- | --- | --- | --- | --- | --- |
| CMJ jump height | Lower-limb power | m | Placebo | 30 min | -0.014 ± 0.016 | -3.11 ± 3.58 |
|  |  |  | Placebo | 60 min | -0.021 ± 0.025 | -4.69 ± 5.83 |
|  |  |  | 3 mg·kg⁻¹ | 30 min | 0.039 ± 0.030 | 9.42 ± 8.05 |
|  |  |  | 3 mg·kg⁻¹ | 60 min | 0.057 ± 0.040 | 13.84 ± 10.90 |
|  |  |  | 6 mg·kg⁻¹ | 30 min | 0.038 ± 0.033 | 9.52 ± 9.01 |
|  |  |  | 6 mg·kg⁻¹ | 60 min | 0.062 ± 0.032 | 14.96 ± 9.17 |
| CMJ peak power | Lower-limb power | W | Placebo | 30 min | -161.0 ± 144.0 | -3.57 ± 2.92 |
|  |  |  | Placebo | 60 min | -174.1 ± 176.1 | -3.77 ± 3.30 |
|  |  |  | 3 mg·kg⁻¹ | 30 min | 249.3 ± 108.2 | 6.20 ± 2.73 |
|  |  |  | 3 mg·kg⁻¹ | 60 min | 451.5 ± 213.3 | 11.29 ± 5.25 |
|  |  |  | 6 mg·kg⁻¹ | 30 min | 228.5 ± 132.0 | 5.74 ± 3.89 |
|  |  |  | 6 mg·kg⁻¹ | 60 min | 383.8 ± 201.1 | 9.59 ± 5.55 |
| CMJ relative peak power | Lower-limb power | W·kg⁻¹ | Placebo | 30 min | -1.87 ± 2.39 | -3.07 ± 3.79 |
|  |  |  | Placebo | 60 min | -1.93 ± 2.60 | -3.13 ± 3.90 |
|  |  |  | 3 mg·kg⁻¹ | 30 min | 3.33 ± 1.54 | 6.28 ± 3.07 |
|  |  |  | 3 mg·kg⁻¹ | 60 min | 6.27 ± 2.94 | 11.76 ± 5.90 |
|  |  |  | 6 mg·kg⁻¹ | 30 min | 3.13 ± 2.20 | 5.97 ± 5.05 |
|  |  |  | 6 mg·kg⁻¹ | 60 min | 5.53 ± 3.23 | 10.17 ± 6.38 |
| BPU concentric impulse | Upper-limb power | N·s | Placebo | 30 min | -1.27 ± 24.63 | 1.01 ± 30.08 |
|  |  |  | Placebo | 60 min | -2.40 ± 11.51 | -2.18 ± 13.79 |
|  |  |  | 3 mg·kg⁻¹ | 30 min | 10.33 ± 13.59 | 14.11 ± 16.66 |
|  |  |  | 3 mg·kg⁻¹ | 60 min | 10.53 ± 14.56 | 13.01 ± 15.93 |
|  |  |  | 6 mg·kg⁻¹ | 30 min | 19.67 ± 19.58 | 26.96 ± 29.63 |
|  |  |  | 6 mg·kg⁻¹ | 60 min | 18.67 ± 18.09 | 27.91 ± 33.07 |
| BPU concentric mean power | Upper-limb power | W | Placebo | 30 min | -14.5 ± 230.8 | 0.62 ± 27.80 |
|  |  |  | Placebo | 60 min | -22.0 ± 111.8 | -1.87 ± 13.00 |
|  |  |  | 3 mg·kg⁻¹ | 30 min | 106.6 ± 130.2 | 13.77 ± 15.34 |
|  |  |  | 3 mg·kg⁻¹ | 60 min | 122.5 ± 127.1 | 14.13 ± 13.22 |
|  |  |  | 6 mg·kg⁻¹ | 30 min | 183.6 ± 237.4 | 24.00 ± 28.17 |
|  |  |  | 6 mg·kg⁻¹ | 60 min | 196.3 ± 180.9 | 26.67 ± 27.58 |
| BPU concentric peak power | Upper-limb power | W | Placebo | 30 min | -39.7 ± 254.3 | -1.17 ± 15.65 |
|  |  |  | Placebo | 60 min | -6.1 ± 154.7 | -0.03 ± 10.86 |
|  |  |  | 3 mg·kg⁻¹ | 30 min | 204.7 ± 118.4 | 15.33 ± 8.99 |
|  |  |  | 3 mg·kg⁻¹ | 60 min | 235.5 ± 186.1 | 16.64 ± 11.94 |
|  |  |  | 6 mg·kg⁻¹ | 30 min | 335.5 ± 382.4 | 25.53 ± 29.24 |
|  |  |  | 6 mg·kg⁻¹ | 60 min | 310.5 ± 311.1 | 25.03 ± 27.21 |
| Shoulder flexion PT | Isokinetic peak torque | N·m | Placebo | 30 min | -6.00 ± 7.79 | -7.66 ± 11.60 |
|  |  |  | Placebo | 60 min | -2.96 ± 11.99 | -2.75 ± 14.20 |
|  |  |  | 3 mg·kg⁻¹ | 30 min | 4.51 ± 11.64 | 9.22 ± 15.97 |
|  |  |  | 3 mg·kg⁻¹ | 60 min | 9.33 ± 10.73 | 15.47 ± 16.86 |
|  |  |  | 6 mg·kg⁻¹ | 30 min | 9.84 ± 16.10 | 16.05 ± 24.83 |
|  |  |  | 6 mg·kg⁻¹ | 60 min | 13.87 ± 18.41 | 22.73 ± 27.17 |
| Shoulder extension PT | Isokinetic peak torque | N·m | Placebo | 30 min | 1.24 ± 8.83 | 1.95 ± 9.97 |
|  |  |  | Placebo | 60 min | -3.84 ± 11.41 | -2.75 ± 10.32 |
|  |  |  | 3 mg·kg⁻¹ | 30 min | 8.11 ± 6.22 | 8.39 ± 8.04 |
|  |  |  | 3 mg·kg⁻¹ | 60 min | 10.02 ± 11.11 | 10.55 ± 12.34 |
|  |  |  | 6 mg·kg⁻¹ | 30 min | 18.84 ± 19.31 | 21.43 ± 26.43 |
|  |  |  | 6 mg·kg⁻¹ | 60 min | 26.71 ± 21.92 | 30.03 ± 32.60 |
| Knee flexion PT | Isokinetic peak torque | N·m | Placebo | 30 min | 2.22 ± 8.63 | 2.47 ± 6.72 |
|  |  |  | Placebo | 60 min | -0.76 ± 14.46 | 0.09 ± 11.83 |
|  |  |  | 3 mg·kg⁻¹ | 30 min | 13.98 ± 18.79 | 14.05 ± 17.37 |
|  |  |  | 3 mg·kg⁻¹ | 60 min | 9.98 ± 26.58 | 11.02 ± 21.33 |
|  |  |  | 6 mg·kg⁻¹ | 30 min | 19.89 ± 14.42 | 18.49 ± 14.98 |
|  |  |  | 6 mg·kg⁻¹ | 60 min | 33.20 ± 17.23 | 30.25 ± 19.10 |
| Knee extension PT | Isokinetic peak torque | N·m | Placebo | 30 min | 1.13 ± 13.93 | 1.19 ± 8.28 |
|  |  |  | Placebo | 60 min | -0.71 ± 14.38 | -0.07 ± 8.98 |
|  |  |  | 3 mg·kg⁻¹ | 30 min | 20.78 ± 34.05 | 13.10 ± 21.15 |
|  |  |  | 3 mg·kg⁻¹ | 60 min | 12.00 ± 30.84 | 8.87 ± 16.81 |
|  |  |  | 6 mg·kg⁻¹ | 30 min | 31.00 ± 15.69 | 19.06 ± 10.31 |
|  |  |  | 6 mg·kg⁻¹ | 60 min | 46.91 ± 22.16 | 28.76 ± 15.90 |

*Absolute and percent changes were calculated relative to the baseline value within the same supplementation condition.*

Supplementary Table S5. Baseline reliability and within-participant variability.

| **Outcome** | **Unit** | **ICC(2,1)** | **ICC(2,1) interpretation** | **Typical error** | **TE CV (%)** | **Mean individual CV (%)** |
| --- | --- | --- | --- | --- | --- | --- |
| BPU concentric impulse | N·s | 0.564 [0.272, 0.803] | moderate | 11.74 | 13.6 | 12.5 |
| BPU concentric mean power | W | 0.671 [0.406, 0.860] | moderate | 104.20 | 11.6 | 10.7 |
| BPU concentric peak power | W | 0.870 [0.689, 0.952] | good | 45.01 | 4.6 | 4.8 |
| CMJ jump height | m | 0.865 [0.625, 0.954] | good | 0.02 | 5.4 | 6.2 |
| CMJ peak power | W | 0.863 [0.569, 0.955] | good | 165.80 | 3.9 | 5.0 |
| CMJ relative peak power | W·kg⁻¹ | 0.813 [0.492, 0.935] | good | 2.51 | 4.4 | 5.8 |
| Knee extension PT | N·m | 0.678 [0.379, 0.868] | moderate | 26.01 | 14.8 | 17.5 |
| Knee flexion PT | N·m | 0.838 [0.667, 0.937] | good | 11.14 | 8.9 | 7.9 |
| Shoulder extension PT | N·m | 0.677 [0.415, 0.863] | moderate | 11.58 | 11.2 | 8.8 |
| Shoulder flexion PT | N·m | 0.800 [0.603, 0.920] | good | 8.64 | 11.5 | 8.1 |

*Reliability was calculated from the three pre-ingestion baseline measurements across the PLA, CAF3, and CAF6 conditions.*

Supplementary Table S6a. Strict individual-response thresholds and responder summary for CMJ and BPU outcomes.

| **Outcome** | **Domain** | **Unit** | **Dose** | **Time** | **SWC** | **TE** | **Strict threshold** | **Positive responders, n (%)** | **Unclear, n (%)** | **Negative responders, n (%)** |
| --- | --- | --- | --- | --- | --- | --- | --- | --- | --- | --- |
| BPU concentric impulse | BPU | N·s | 3 mg·kg⁻¹ | 30 min | 3.439 | 11.743 | 11.743 | 9 (60.0) | 3 (20.0) | 3 (20.0) |
|  |  |  | 3 mg·kg⁻¹ | 60 min |  |  |  | 10 (66.7) | 2 (13.3) | 3 (20.0) |
|  |  |  | 6 mg·kg⁻¹ | 30 min |  |  |  | 9 (60.0) | 5 (33.3) | 1 (6.7) |
|  |  |  | 6 mg·kg⁻¹ | 60 min |  |  |  | 8 (53.3) | 7 (46.7) | 0 (0.0) |
| BPU concentric mean power | BPU | W | 3 mg·kg⁻¹ | 30 min | 34.979 | 104.2 | 104.2 | 9 (60.0) | 2 (13.3) | 4 (26.7) |
|  |  |  | 3 mg·kg⁻¹ | 60 min |  |  |  | 11 (73.3) | 1 (6.7) | 3 (20.0) |
|  |  |  | 6 mg·kg⁻¹ | 30 min |  |  |  | 10 (66.7) | 3 (20.0) | 2 (13.3) |
|  |  |  | 6 mg·kg⁻¹ | 60 min |  |  |  | 9 (60.0) | 6 (40.0) | 0 (0.0) |
| BPU concentric peak power | BPU | W | 3 mg·kg⁻¹ | 30 min | 25.259 | 45.009 | 45.009 | 14 (93.3) | 1 (6.7) | 0 (0.0) |
|  |  |  | 3 mg·kg⁻¹ | 60 min |  |  |  | 14 (93.3) | 1 (6.7) | 0 (0.0) |
|  |  |  | 6 mg·kg⁻¹ | 30 min |  |  |  | 14 (93.3) | 1 (6.7) | 0 (0.0) |
|  |  |  | 6 mg·kg⁻¹ | 60 min |  |  |  | 14 (93.3) | 1 (6.7) | 0 (0.0) |
| CMJ jump height | CMJ | m | 3 mg·kg⁻¹ | 30 min | 0.016 | 0.024 | 0.024 | 15 (100.0) | 0 (0.0) | 0 (0.0) |
|  |  |  | 3 mg·kg⁻¹ | 60 min |  |  |  | 15 (100.0) | 0 (0.0) | 0 (0.0) |
|  |  |  | 6 mg·kg⁻¹ | 30 min |  |  |  | 13 (86.7) | 2 (13.3) | 0 (0.0) |
|  |  |  | 6 mg·kg⁻¹ | 60 min |  |  |  | 15 (100.0) | 0 (0.0) | 0 (0.0) |
| CMJ peak power | CMJ | W | 3 mg·kg⁻¹ | 30 min | 122.2 | 165.8 | 165.8 | 12 (80.0) | 3 (20.0) | 0 (0.0) |
|  |  |  | 3 mg·kg⁻¹ | 60 min |  |  |  | 13 (86.7) | 2 (13.3) | 0 (0.0) |
|  |  |  | 6 mg·kg⁻¹ | 30 min |  |  |  | 13 (86.7) | 2 (13.3) | 0 (0.0) |
|  |  |  | 6 mg·kg⁻¹ | 60 min |  |  |  | 15 (100.0) | 0 (0.0) | 0 (0.0) |
| CMJ relative peak power | CMJ | W·kg⁻¹ | 3 mg·kg⁻¹ | 30 min | 1.400 | 2.515 | 2.515 | 12 (80.0) | 3 (20.0) | 0 (0.0) |
|  |  |  | 3 mg·kg⁻¹ | 60 min |  |  |  | 14 (93.3) | 1 (6.7) | 0 (0.0) |
|  |  |  | 6 mg·kg⁻¹ | 30 min |  |  |  | 12 (80.0) | 3 (20.0) | 0 (0.0) |
|  |  |  | 6 mg·kg⁻¹ | 60 min |  |  |  | 15 (100.0) | 0 (0.0) | 0 (0.0) |

*Strict threshold = max(SWC, TE). Positive, unclear, and negative classifications were based on placebo-adjusted changes.*

Supplementary Table S6b. Strict individual-response thresholds and responder summary for isokinetic peak torque outcomes.

| **Outcome** | **Domain** | **Unit** | **Dose** | **Time** | **SWC** | **TE** | **Strict threshold** | **Positive responders, n (%)** | **Unclear, n (%)** | **Negative responders, n (%)** |
| --- | --- | --- | --- | --- | --- | --- | --- | --- | --- | --- |
| Knee extension PT | Isokinetic peak torque | N·m | 3 mg·kg⁻¹ | 30 min | 8.957 | 26.011 | 26.011 | 7 (46.7) | 7 (46.7) | 1 (6.7) |
|  |  |  | 3 mg·kg⁻¹ | 60 min |  |  |  | 5 (33.3) | 8 (53.3) | 2 (13.3) |
|  |  |  | 6 mg·kg⁻¹ | 30 min |  |  |  | 9 (60.0) | 6 (40.0) | 0 (0.0) |
|  |  |  | 6 mg·kg⁻¹ | 60 min |  |  |  | 11 (73.3) | 4 (26.7) | 0 (0.0) |
| Knee flexion PT | Isokinetic peak torque | N·m | 3 mg·kg⁻¹ | 30 min | 5.007 | 11.138 | 11.138 | 9 (60.0) | 5 (33.3) | 1 (6.7) |
|  |  |  | 3 mg·kg⁻¹ | 60 min |  |  |  | 10 (66.7) | 3 (20.0) | 2 (13.3) |
|  |  |  | 6 mg·kg⁻¹ | 30 min |  |  |  | 10 (66.7) | 4 (26.7) | 1 (6.7) |
|  |  |  | 6 mg·kg⁻¹ | 60 min |  |  |  | 14 (93.3) | 1 (6.7) | 0 (0.0) |
| Shoulder extension PT | Isokinetic peak torque | N·m | 3 mg·kg⁻¹ | 30 min | 3.312 | 11.577 | 11.577 | 3 (20.0) | 12 (80.0) | 0 (0.0) |
|  |  |  | 3 mg·kg⁻¹ | 60 min |  |  |  | 9 (60.0) | 6 (40.0) | 0 (0.0) |
|  |  |  | 6 mg·kg⁻¹ | 30 min |  |  |  | 8 (53.3) | 6 (40.0) | 1 (6.7) |
|  |  |  | 6 mg·kg⁻¹ | 60 min |  |  |  | 12 (80.0) | 3 (20.0) | 0 (0.0) |
| Shoulder flexion PT | Isokinetic peak torque | N·m | 3 mg·kg⁻¹ | 30 min | 3.450 | 8.641 | 8.641 | 7 (46.7) | 7 (46.7) | 1 (6.7) |
|  |  |  | 3 mg·kg⁻¹ | 60 min |  |  |  | 10 (66.7) | 4 (26.7) | 1 (6.7) |
|  |  |  | 6 mg·kg⁻¹ | 30 min |  |  |  | 11 (73.3) | 3 (20.0) | 1 (6.7) |
|  |  |  | 6 mg·kg⁻¹ | 60 min |  |  |  | 12 (80.0) | 1 (6.7) | 2 (13.3) |

*Strict threshold = max(SWC, TE). Positive, unclear, and negative classifications were based on placebo-adjusted changes.*

Supplementary Table S7. Domain-level responder summary using the strict threshold.

| **Domain** | **Dose** | **Time** | **Outcome-level observations** | **Positive responders, n (%)** | **Unclear, n (%)** | **Negative responders, n (%)** |
| --- | --- | --- | --- | --- | --- | --- |
| BPU | 3 mg·kg⁻¹ | 30 min | 45 | 32 (71.1) | 6 (13.3) | 7 (15.6) |
|  | 3 mg·kg⁻¹ | 60 min | 45 | 35 (77.8) | 4 (8.9) | 6 (13.3) |
|  | 6 mg·kg⁻¹ | 30 min | 45 | 33 (73.3) | 9 (20.0) | 3 (6.7) |
|  | 6 mg·kg⁻¹ | 60 min | 45 | 31 (68.9) | 14 (31.1) | 0 (0.0) |
| CMJ | 3 mg·kg⁻¹ | 30 min | 45 | 39 (86.7) | 6 (13.3) | 0 (0.0) |
|  | 3 mg·kg⁻¹ | 60 min | 45 | 42 (93.3) | 3 (6.7) | 0 (0.0) |
|  | 6 mg·kg⁻¹ | 30 min | 45 | 38 (84.4) | 7 (15.6) | 0 (0.0) |
|  | 6 mg·kg⁻¹ | 60 min | 45 | 45 (100.0) | 0 (0.0) | 0 (0.0) |
| Isokinetic peak torque | 3 mg·kg⁻¹ | 30 min | 60 | 26 (43.3) | 31 (51.7) | 3 (5.0) |
|  | 3 mg·kg⁻¹ | 60 min | 60 | 34 (56.7) | 21 (35.0) | 5 (8.3) |
|  | 6 mg·kg⁻¹ | 30 min | 60 | 38 (63.3) | 19 (31.7) | 3 (5.0) |
|  | 6 mg·kg⁻¹ | 60 min | 60 | 49 (81.7) | 9 (15.0) | 2 (3.3) |

*Outcome-level observations represent participants × outcomes within each domain. CMJ contains 3 outcomes, BPU contains 3 outcomes, and isokinetic peak torque contains 4 outcomes.*

Supplementary Table S8. Co-response across CAF3 and CAF6 at the same time point.

| **Outcome** | **Domain** | **Unit** | **Time** | **n** | **CAF3 responders** | **CAF6 responders** | **Co-responders CAF3+CAF6** | **Co-response (%)** |
| --- | --- | --- | --- | --- | --- | --- | --- | --- |
| BPU concentric impulse | BPU | N·s | 30 min | 15 | 9 | 9 | 8 | 53.3 |
|  |  |  | 60 min | 15 | 10 | 8 | 6 | 40.0 |
| BPU concentric mean power | BPU | W | 30 min | 15 | 9 | 10 | 8 | 53.3 |
|  |  |  | 60 min | 15 | 11 | 9 | 8 | 53.3 |
| BPU concentric peak power | BPU | W | 30 min | 15 | 14 | 14 | 13 | 86.7 |
|  |  |  | 60 min | 15 | 14 | 14 | 13 | 86.7 |
| CMJ jump height | CMJ | m | 30 min | 15 | 15 | 13 | 13 | 86.7 |
|  |  |  | 60 min | 15 | 15 | 15 | 15 | 100.0 |
| CMJ peak power | CMJ | W | 30 min | 15 | 12 | 13 | 12 | 80.0 |
|  |  |  | 60 min | 15 | 13 | 15 | 13 | 86.7 |
| CMJ relative peak power | CMJ | W·kg⁻¹ | 30 min | 15 | 12 | 12 | 11 | 73.3 |
|  |  |  | 60 min | 15 | 14 | 15 | 14 | 93.3 |
| Knee extension PT | Isokinetic peak torque | N·m | 30 min | 15 | 7 | 9 | 4 | 26.7 |
|  |  |  | 60 min | 15 | 5 | 11 | 5 | 33.3 |
| Knee flexion PT | Isokinetic peak torque | N·m | 30 min | 15 | 9 | 10 | 5 | 33.3 |
|  |  |  | 60 min | 15 | 10 | 14 | 10 | 66.7 |
| Shoulder extension PT | Isokinetic peak torque | N·m | 30 min | 15 | 3 | 8 | 3 | 20.0 |
|  |  |  | 60 min | 15 | 9 | 12 | 8 | 53.3 |
| Shoulder flexion PT | Isokinetic peak torque | N·m | 30 min | 15 | 7 | 11 | 6 | 40.0 |
|  |  |  | 60 min | 15 | 10 | 12 | 9 | 60.0 |

*Co-responders were participants classified as positive responders under both CAF3 and CAF6 for the same outcome and post-ingestion time point.*

Supplementary Table S9. Co-response across 30 and 60 min within the same caffeine dose.

| **Outcome** | **Domain** | **Unit** | **Dose** | **n** | **30-min responders** | **60-min responders** | **Co-responders 30+60 min** | **Co-response (%)** |
| --- | --- | --- | --- | --- | --- | --- | --- | --- |
| BPU concentric impulse | BPU | N·s | 3 mg·kg⁻¹ | 15 | 9 | 10 | 7 | 46.7 |
|  |  |  | 6 mg·kg⁻¹ | 15 | 9 | 8 | 7 | 46.7 |
| BPU concentric mean power | BPU | W | 3 mg·kg⁻¹ | 15 | 9 | 11 | 8 | 53.3 |
|  |  |  | 6 mg·kg⁻¹ | 15 | 10 | 9 | 9 | 60.0 |
| BPU concentric peak power | BPU | W | 3 mg·kg⁻¹ | 15 | 14 | 14 | 13 | 86.7 |
|  |  |  | 6 mg·kg⁻¹ | 15 | 14 | 14 | 13 | 86.7 |
| CMJ jump height | CMJ | m | 3 mg·kg⁻¹ | 15 | 15 | 15 | 15 | 100.0 |
|  |  |  | 6 mg·kg⁻¹ | 15 | 13 | 15 | 13 | 86.7 |
| CMJ peak power | CMJ | W | 3 mg·kg⁻¹ | 15 | 12 | 13 | 12 | 80.0 |
|  |  |  | 6 mg·kg⁻¹ | 15 | 13 | 15 | 13 | 86.7 |
| CMJ relative peak power | CMJ | W·kg⁻¹ | 3 mg·kg⁻¹ | 15 | 12 | 14 | 12 | 80.0 |
|  |  |  | 6 mg·kg⁻¹ | 15 | 12 | 15 | 12 | 80.0 |
| Knee extension PT | Isokinetic peak torque | N·m | 3 mg·kg⁻¹ | 15 | 7 | 5 | 5 | 33.3 |
|  |  |  | 6 mg·kg⁻¹ | 15 | 9 | 11 | 8 | 53.3 |
| Knee flexion PT | Isokinetic peak torque | N·m | 3 mg·kg⁻¹ | 15 | 9 | 10 | 6 | 40.0 |
|  |  |  | 6 mg·kg⁻¹ | 15 | 10 | 14 | 9 | 60.0 |
| Shoulder extension PT | Isokinetic peak torque | N·m | 3 mg·kg⁻¹ | 15 | 3 | 9 | 3 | 20.0 |
|  |  |  | 6 mg·kg⁻¹ | 15 | 8 | 12 | 7 | 46.7 |
| Shoulder flexion PT | Isokinetic peak torque | N·m | 3 mg·kg⁻¹ | 15 | 7 | 10 | 7 | 46.7 |
|  |  |  | 6 mg·kg⁻¹ | 15 | 11 | 12 | 10 | 66.7 |

*Co-responders were participants classified as positive responders at both 30 and 60 min for the same outcome and caffeine dose.*

Supplementary Table S10. Subject-level response profile across all outcomes using the strict threshold.

| **Participant** | **Dose** | **Time** | **Outcomes** | **Positive** | **Unclear** | **Negative** | **Positive response rate (%)** | **Negative response rate (%)** |
| --- | --- | --- | --- | --- | --- | --- | --- | --- |
| 1 | 3 mg·kg⁻¹ | 30 min | 10 | 6 | 2 | 2 | 60.0 | 20.0 |
|  | 3 mg·kg⁻¹ | 60 min | 10 | 10 | 0 | 0 | 100.0 | 0.0 |
|  | 6 mg·kg⁻¹ | 30 min | 10 | 7 | 2 | 1 | 70.0 | 10.0 |
|  | 6 mg·kg⁻¹ | 60 min | 10 | 7 | 3 | 0 | 70.0 | 0.0 |
| 2 | 3 mg·kg⁻¹ | 30 min | 10 | 5 | 4 | 1 | 50.0 | 10.0 |
|  | 3 mg·kg⁻¹ | 60 min | 10 | 7 | 2 | 1 | 70.0 | 10.0 |
|  | 6 mg·kg⁻¹ | 30 min | 10 | 6 | 4 | 0 | 60.0 | 0.0 |
|  | 6 mg·kg⁻¹ | 60 min | 10 | 8 | 2 | 0 | 80.0 | 0.0 |
| 3 | 3 mg·kg⁻¹ | 30 min | 10 | 7 | 3 | 0 | 70.0 | 0.0 |
|  | 3 mg·kg⁻¹ | 60 min | 10 | 8 | 2 | 0 | 80.0 | 0.0 |
|  | 6 mg·kg⁻¹ | 30 min | 10 | 8 | 1 | 1 | 80.0 | 10.0 |
|  | 6 mg·kg⁻¹ | 60 min | 10 | 9 | 0 | 1 | 90.0 | 10.0 |
| 4 | 3 mg·kg⁻¹ | 30 min | 10 | 6 | 4 | 0 | 60.0 | 0.0 |
|  | 3 mg·kg⁻¹ | 60 min | 10 | 6 | 2 | 2 | 60.0 | 20.0 |
|  | 6 mg·kg⁻¹ | 30 min | 10 | 5 | 5 | 0 | 50.0 | 0.0 |
|  | 6 mg·kg⁻¹ | 60 min | 10 | 8 | 2 | 0 | 80.0 | 0.0 |
| 5 | 3 mg·kg⁻¹ | 30 min | 10 | 6 | 4 | 0 | 60.0 | 0.0 |
|  | 3 mg·kg⁻¹ | 60 min | 10 | 7 | 2 | 1 | 70.0 | 10.0 |
|  | 6 mg·kg⁻¹ | 30 min | 10 | 7 | 3 | 0 | 70.0 | 0.0 |
|  | 6 mg·kg⁻¹ | 60 min | 10 | 10 | 0 | 0 | 100.0 | 0.0 |
| 6 | 3 mg·kg⁻¹ | 30 min | 10 | 8 | 2 | 0 | 80.0 | 0.0 |
|  | 3 mg·kg⁻¹ | 60 min | 10 | 7 | 2 | 1 | 70.0 | 10.0 |
|  | 6 mg·kg⁻¹ | 30 min | 10 | 8 | 2 | 0 | 80.0 | 0.0 |
|  | 6 mg·kg⁻¹ | 60 min | 10 | 7 | 3 | 0 | 70.0 | 0.0 |
| 7 | 3 mg·kg⁻¹ | 30 min | 10 | 8 | 2 | 0 | 80.0 | 0.0 |
|  | 3 mg·kg⁻¹ | 60 min | 10 | 6 | 2 | 2 | 60.0 | 20.0 |
|  | 6 mg·kg⁻¹ | 30 min | 10 | 9 | 1 | 0 | 90.0 | 0.0 |
|  | 6 mg·kg⁻¹ | 60 min | 10 | 8 | 2 | 0 | 80.0 | 0.0 |
| 8 | 3 mg·kg⁻¹ | 30 min | 10 | 9 | 1 | 0 | 90.0 | 0.0 |
|  | 3 mg·kg⁻¹ | 60 min | 10 | 9 | 1 | 0 | 90.0 | 0.0 |
|  | 6 mg·kg⁻¹ | 30 min | 10 | 6 | 4 | 0 | 60.0 | 0.0 |
|  | 6 mg·kg⁻¹ | 60 min | 10 | 7 | 3 | 0 | 70.0 | 0.0 |
| 9 | 3 mg·kg⁻¹ | 30 min | 10 | 4 | 5 | 1 | 40.0 | 10.0 |
|  | 3 mg·kg⁻¹ | 60 min | 10 | 8 | 2 | 0 | 80.0 | 0.0 |
|  | 6 mg·kg⁻¹ | 30 min | 10 | 6 | 4 | 0 | 60.0 | 0.0 |
|  | 6 mg·kg⁻¹ | 60 min | 10 | 10 | 0 | 0 | 100.0 | 0.0 |
| 10 | 3 mg·kg⁻¹ | 30 min | 10 | 7 | 1 | 2 | 70.0 | 20.0 |
|  | 3 mg·kg⁻¹ | 60 min | 10 | 6 | 2 | 2 | 60.0 | 20.0 |
|  | 6 mg·kg⁻¹ | 30 min | 10 | 7 | 3 | 0 | 70.0 | 0.0 |
|  | 6 mg·kg⁻¹ | 60 min | 10 | 6 | 4 | 0 | 60.0 | 0.0 |
| 11 | 3 mg·kg⁻¹ | 30 min | 10 | 8 | 0 | 2 | 80.0 | 20.0 |
|  | 3 mg·kg⁻¹ | 60 min | 10 | 8 | 2 | 0 | 80.0 | 0.0 |
|  | 6 mg·kg⁻¹ | 30 min | 10 | 8 | 2 | 0 | 80.0 | 0.0 |
|  | 6 mg·kg⁻¹ | 60 min | 10 | 9 | 1 | 0 | 90.0 | 0.0 |
| 12 | 3 mg·kg⁻¹ | 30 min | 10 | 5 | 3 | 2 | 50.0 | 20.0 |
|  | 3 mg·kg⁻¹ | 60 min | 10 | 6 | 2 | 2 | 60.0 | 20.0 |
|  | 6 mg·kg⁻¹ | 30 min | 10 | 4 | 3 | 3 | 40.0 | 30.0 |
|  | 6 mg·kg⁻¹ | 60 min | 10 | 7 | 2 | 1 | 70.0 | 10.0 |
| 13 | 3 mg·kg⁻¹ | 30 min | 10 | 4 | 6 | 0 | 40.0 | 0.0 |
|  | 3 mg·kg⁻¹ | 60 min | 10 | 8 | 2 | 0 | 80.0 | 0.0 |
|  | 6 mg·kg⁻¹ | 30 min | 10 | 10 | 0 | 0 | 100.0 | 0.0 |
|  | 6 mg·kg⁻¹ | 60 min | 10 | 9 | 1 | 0 | 90.0 | 0.0 |
| 14 | 3 mg·kg⁻¹ | 30 min | 10 | 6 | 4 | 0 | 60.0 | 0.0 |
|  | 3 mg·kg⁻¹ | 60 min | 10 | 6 | 4 | 0 | 60.0 | 0.0 |
|  | 6 mg·kg⁻¹ | 30 min | 10 | 10 | 0 | 0 | 100.0 | 0.0 |
|  | 6 mg·kg⁻¹ | 60 min | 10 | 10 | 0 | 0 | 100.0 | 0.0 |
| 15 | 3 mg·kg⁻¹ | 30 min | 10 | 8 | 2 | 0 | 80.0 | 0.0 |
|  | 3 mg·kg⁻¹ | 60 min | 10 | 9 | 1 | 0 | 90.0 | 0.0 |
|  | 6 mg·kg⁻¹ | 30 min | 10 | 8 | 1 | 1 | 80.0 | 10.0 |
|  | 6 mg·kg⁻¹ | 60 min | 10 | 10 | 0 | 0 | 100.0 | 0.0 |

*Each row summarizes one participant's response classification across all 10 outcomes for a given caffeine dose and time point.*

Supplementary Table S11. Key Bonferroni-adjusted pairwise comparisons.

| **Outcome** | **Domain** | **Comparison type** | **Time** | **Dose** | **Contrast** | **Estimate** | **SE** | **df** | **t** | **Bonferroni-adjusted P** |
| --- | --- | --- | --- | --- | --- | --- | --- | --- | --- | --- |
| CMJ jump height | Lower-limb power | Dose within each time | 60 min |  | Placebo - 3 mg·kg⁻¹ | -0.039 | 0.009 | 14 | -4.110 | 0.003 |
|  |  | Dose within each time | 60 min |  | Placebo - 6 mg·kg⁻¹ | -0.051 | 0.009 | 14 | -5.584 | < 0.001 |
|  |  | Time within each dose |  | Placebo | Baseline - 60 min | 0.021 | 0.006 | 14 | 3.238 | 0.018 |
|  |  | Time within each dose |  | 3 mg·kg⁻¹ | Baseline - 60 min | -0.057 | 0.010 | 14 | -5.527 | < 0.001 |
|  |  | Time within each dose |  | 6 mg·kg⁻¹ | Baseline - 60 min | -0.062 | 0.008 | 14 | -7.445 | < 0.001 |
| CMJ peak power | Lower-limb power | Dose within each time | 60 min |  | Placebo - 3 mg·kg⁻¹ | -279.200 | 64.700 | 14 | -4.315 | 0.002 |
|  |  | Dose within each time | 60 min |  | Placebo - 6 mg·kg⁻¹ | -348.300 | 59.200 | 14 | -5.884 | < 0.001 |
|  |  | Time within each dose |  | Placebo | Baseline - 60 min | 174.100 | 45.500 | 14 | 3.831 | 0.006 |
|  |  | Time within each dose |  | 3 mg·kg⁻¹ | Baseline - 60 min | -451.500 | 55.100 | 14 | -8.200 | < 0.001 |
|  |  | Time within each dose |  | 6 mg·kg⁻¹ | Baseline - 60 min | -383.800 | 51.900 | 14 | -7.390 | < 0.001 |
| CMJ relative peak power | Lower-limb power | Dose within each time | 60 min |  | Placebo - 3 mg·kg⁻¹ | -3.600 | 0.930 | 14 | -3.871 | 0.005 |
|  |  | Dose within each time | 60 min |  | Placebo - 6 mg·kg⁻¹ | -4.470 | 0.970 | 14 | -4.603 | 0.001 |
|  |  | Time within each dose |  | Placebo | Baseline - 60 min | 1.930 | 0.670 | 14 | 2.875 | 0.037 |
|  |  | Time within each dose |  | 3 mg·kg⁻¹ | Baseline - 60 min | -6.270 | 0.760 | 14 | -8.258 | < 0.001 |
|  |  | Time within each dose |  | 6 mg·kg⁻¹ | Baseline - 60 min | -5.530 | 0.830 | 14 | -6.642 | < 0.001 |
| BPU concentric impulse | Upper-limb power | Dose within each time | 60 min |  | Placebo - 6 mg·kg⁻¹ | -12.200 | 4.280 | 14 | -2.854 | 0.038 |
|  |  | Time within each dose |  | 3 mg·kg⁻¹ | Baseline - 60 min | -10.530 | 3.760 | 14 | -2.802 | 0.042 |
|  |  | Time within each dose |  | 6 mg·kg⁻¹ | Baseline - 60 min | -18.670 | 4.670 | 14 | -3.997 | 0.004 |
| BPU concentric mean power | Upper-limb power | Dose within each time | 60 min |  | Placebo - 6 mg·kg⁻¹ | -137.300 | 44.500 | 14 | -3.084 | 0.024 |
|  |  | Time within each dose |  | 3 mg·kg⁻¹ | Baseline - 60 min | -122.500 | 32.800 | 14 | -3.732 | 0.007 |
|  |  | Time within each dose |  | 6 mg·kg⁻¹ | Baseline - 60 min | -196.300 | 46.700 | 14 | -4.204 | 0.003 |
| BPU concentric peak power | Upper-limb power | Dose within each time | 60 min |  | Placebo - 6 mg·kg⁻¹ | -220.700 | 65.700 | 14 | -3.357 | 0.014 |
|  |  | Time within each dose |  | 3 mg·kg⁻¹ | Baseline - 60 min | -235.500 | 48.000 | 14 | -4.902 | < 0.001 |
|  |  | Time within each dose |  | 6 mg·kg⁻¹ | Baseline - 60 min | -310.500 | 80.300 | 14 | -3.865 | 0.005 |
| Shoulder flexion PT | Isokinetic peak torque | Dose within each time | 60 min |  | Placebo - 3 mg·kg⁻¹ | -9.070 | 2.270 | 14 | -3.991 | 0.004 |
|  |  | Dose within each time | 60 min |  | Placebo - 6 mg·kg⁻¹ | -16.330 | 4.210 | 14 | -3.880 | 0.005 |
|  |  | Time within each dose |  | 3 mg·kg⁻¹ | Baseline - 60 min | -9.330 | 2.770 | 14 | -3.368 | 0.014 |
|  |  | Time within each dose |  | 6 mg·kg⁻¹ | Baseline - 60 min | -13.870 | 4.750 | 14 | -2.917 | 0.034 |
| Shoulder extension PT | Isokinetic peak torque | Dose within each time | 60 min |  | Placebo - 3 mg·kg⁻¹ | -13.440 | 4.640 | 14 | -2.895 | 0.035 |
|  |  | Dose within each time | 60 min |  | Placebo - 6 mg·kg⁻¹ | -23.820 | 4.200 | 14 | -5.677 | < 0.001 |
|  |  | Time within each dose |  | 3 mg·kg⁻¹ | Baseline - 60 min | -10.020 | 2.870 | 14 | -3.495 | 0.011 |
|  |  | Time within each dose |  | 6 mg·kg⁻¹ | Baseline - 60 min | -26.710 | 5.660 | 14 | -4.721 | < 0.001 |
| Knee flexion PT | Isokinetic peak torque | Dose within each time | 60 min |  | Placebo - 6 mg·kg⁻¹ | -25.780 | 4.450 | 14 | -5.799 | < 0.001 |
|  |  | Time within each dose |  | 6 mg·kg⁻¹ | Baseline - 60 min | -33.200 | 4.450 | 14 | -7.462 | < 0.001 |
| Knee extension PT | Isokinetic peak torque | Dose within each time | 60 min |  | Placebo - 6 mg·kg⁻¹ | -41.360 | 5.780 | 14 | -7.157 | < 0.001 |
|  |  | Dose within each time | 60 min |  | 3 mg·kg⁻¹ - 6 mg·kg⁻¹ | -25.980 | 8.260 | 14 | -3.145 | 0.021 |
|  |  | Time within each dose |  | 6 mg·kg⁻¹ | Baseline - 60 min | -46.910 | 5.720 | 14 | -8.201 | < 0.001 |

*This table reports the key pairwise comparisons supporting the main results. The complete pairwise-comparison output is available in the accompanying Excel workbook.* *P values were Bonferroni-adjusted separately within each outcome-specific post-hoc family; no single global adjustment was applied across pairwise comparisons from different outcomes.*

Supplementary Figures

Supplementary Figure S1. Individual placebo-adjusted responses based on the strict SWC/TE threshold.

Green indicates positive responders, gray indicates trivial/non-responders, and red indicates negative responders. Panels are separated by caffeine dose and post-ingestion time point.

Supplementary Figure S2. Responder rates for placebo-adjusted changes.

Bars show the percentage of participants classified as positive responders under CAF3 and CAF6 at 30 and 60 min for each outcome.

Supplementary Figure S3. Subject-level responder profile across all outcomes.

Bars show the percentage of all assessed outcomes for which each participant was classified as a positive responder under each caffeine dose and time point.
